# Modulating Macrophage–Nucleus Pulposus Cell Crosstalk via Sequential Drug Delivery Attenuates Disc Degeneration

**DOI:** 10.1002/advs.202507178

**Published:** 2025-06-30

**Authors:** Xiangzhen Kong, Rui Hu, Peng Zhang, Qi Li, Kangle Song, Hanwen Gu, Jihui Zheng, Shen Liu, Liang Liu, Lin Jin, Lei Cheng, Chengwei Li, Zhijian Wei

**Affiliations:** ^1^ Department of Orthopaedic Surgery, Qilu Hospital, Cheeloo College of Medicine Shandong University Jinan Shandong 250012 P. R. China; ^2^ Qilu Hospital of Shandong University Jinan Shandong 250012 P. R. China; ^3^ Department of Orthopedics Traditional Chinese Medicine‐Western Medicine Hospital of Cangzhou City Cangzhou 061000 P. R. China; ^4^ Hebei Key Laboratory of Integrated Traditional and Western Medicine in Osteoarthrosis Research (Preparing) Cangzhou 061000 P. R. China; ^5^ Department of Orthopedics International Science and Technology Cooperation Base of Spinal Cord Injury Tianjin Key Laboratory of Spine and Spinal Cord Injury Tianjin Medical University General Hospital No. 154 Anshan Road, Heping District Tianjin 300052 P. R. China; ^6^ Department of Orthopedics Beijing Luhe Hospital Capital Medical University Beijing 101100 P. R. China; ^7^ International Joint Research Laboratory for Biomedical Nanomaterials of Henan Zhoukou Normal University Zhoukou 466001 P. R. China; ^8^ School of Agriculture Sciences Zhengzhou University Zhengzhou 450001 P. R. China; ^9^ Department of Orthopaedicsm The Second Hospital of Shandong University No. 247 Beiyuan Street, Tianqiao District Jinan 250033 P. R. China

**Keywords:** extracellular matrix, hydrogel, intervertebral disc degeneration, macrophage polarization, nanospheres

## Abstract

Intervertebral disc degeneration (IDD) is characterized by an inflammatory environment and dysregulation of the extracellular matrix metabolism (ECM). Improving nucleus pulposus cells (NPCs) condition first requires a supportive surrounding environment. However, how to provide an excellent microenvironment before regulating the function of the NPCs is not yet a complete strategy. In this study, a sequential drug delivery system is comprised of chitosan hydrogel (G‐CS) loaded with Angiotensin (1‐7) and mesoporous silica nanospheres (MSN) coating with α‐mangostin to achieve synergistic effects of anti‐inflammatory and regulation of metabolic disorders of the ECM in NPCs. The system first explosively releases outer G‐CSloaded Angiotensin (1‐7) by exploiting the different solubility characteristics of the two drugs, which improves the inflammatory environment by inhibiting the integrated stress response and regulating the macrophage phenotype. During the second stage, the inner layer of the MSN controlled‐release loaded α‐mangostin to regulate ECM metabolism and synthesis, slowing cell aging and apoptosis. Furthermore, α‐mangostin promotes mitophagy in NPCs by inhibiting the PI3K/AKT/mTOR pathway and activating the Pink1/Parkin pathway, promoting the clearance of damaged mitochondria. The proposed drug delivery system represents an innovative and promising strategy for treating IDD.

## Graphical abstract

The G‐CS/MSN drug delivery system enhances intervertebral disc repair through sequential drug release.

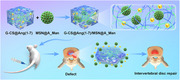

## 1 Introduction

According to the Global Burden of Disease Study, lower back pain (LBP) has remained the leading cause of disability for several years.^[^ ^1^ ^]^ The widespread occurrence of LBP is associated with diminished quality of life and increased medical expenditures, which significantly strain healthcare resources.^[^ ^2^ ^]^ Intervertebral disc degeneration (IDD) is the primary cause of LBP.^[^ ^3^ ^]^

The healthy intervertebral disc (IVD) is a relatively sealed avascular fibrocartilaginous structure The nucleus pulposus (NP) undergoes dehydration and degeneration, while the annulus fibrosus becomes increasingly susceptible to rupture under repetitive mechanical stress with aging. This process has facilitated the invasion of blood vessels and the infiltration of inflammatory cells, such as macrophages, accompanied by the release of inflammatory mediators.^[^ ^4^ ^]^ The inflammatory microenvironment has a unique complexity in IVD, which is accompanied by compromised ECM synthesis, cellular senescence, and dysfunction. However, the treatment of IDD has mostly been carried out simultaneously with anti‐inflammatory and cell function restoration in the past. A combination of therapeutic strategies is required to restore IVD, and the sequential effects of treatments must be carefully considered. Since anabolic balance relies on the presence of healthy NPCs, cell viability is significantly influenced by the microenvironment,^[^ ^5^ ^]^ An increasing number of drug studies have paid attention to the impact of sequential delivery on treatment outcomes recently.^[^ ^6^ ^]^ Exploring IVD regeneration also has gradually begun to focus on step‐by‐step treatment strategies that (i) counteract inflammation in the microenvironment and (ii) enhance NPC activity, preserve cell function, and improve ECM metabolism in a healthy microenvironment. In this study, we integrate drugs to achieve the aforementioned objectives and develop a nanomaterialbased system for their sequential release.

Macrophages, which originate from adult bone marrow hematopoietic stem cells, infiltrate the IVD when it ruptures and is exposed to the immune system, affecting the course of IDD.^[^ ^7^ ^]^ Therefore, selectively promoting the polarization of macrophages and improving the inflammatory microenvironment is crucial for mitigating IDD progression. Angiotensin (1‐7) (Ang (1‐7)) is an endogenous heptapeptide of the renin‐angiotensin system. Previous studies have demonstrated its crucial role in modulating inflammatory responses under various pathological conditions, including colitis, pulmonary fibrosis, hepatic steatosis, and ischemic stroke.^[^ ^8^ ^]^ Moreover, Ang (1‐7) can promote the M2 polarization of microglia/macrophages.^[^ ^9^ ^]^ We hypothesize that Ang (1‐7) administration may improve the IVD microenvironment by modulating macrophage polarization. α‐mangostin (A_Man) is a xanthone derivative isolated from mangosteen peel extract that exhibits a range of pharmacological activities, including antibacterial, antifungal, anti‐inflammatory, antiallergic, antioxidant, and anticancer properties.^[^ ^10^ ^]^ A_Man can protect lipopolysaccharide (LPS)‐stimulated NPCs from NLRP3 inflammasome‐mediated apoptosis by modulating the NF‐κB pathway.^[^ ^11^ ^]^ We have confirmed in our experiments that A_Man can alleviate ECM metabolism dysregulation in the NPCs. And combine A_Man with Ang (1‐7) to inhibit IDD progression.

Hydrogels and mesoporous silica nanoparticles (MSN) are biomaterials commonly used for in vivo drug delivery. Chitosan‐based hydrogels offer several advantages, including a 3D network structure, appropriate degradation rates, and high water content.^[^ ^12^ ^]^ Additionally, chitosan possesses unique adhesive and oxygen‐permeable properties and is nontoxic and biodegradable,^[^ ^13^ ^]^ boasting significant potential for development in the medical field. MSN offers a large specific surface area and pore volume, controllable particle size, and good biocompatibility.^[^ ^14^ ^]^ Additionally, silica can facilitate sustained drug release by adjusting the pore structure and particle size of the carrier.^[^ ^15^ ^]^ Finally, silica nanoparticles can enhance solubility by offering a larger surface area, which improves permeability across cell membranes,^[^ ^16^ ^]^ providing controlled release systems with options for passive or active targeting.

**Scheme 1:**
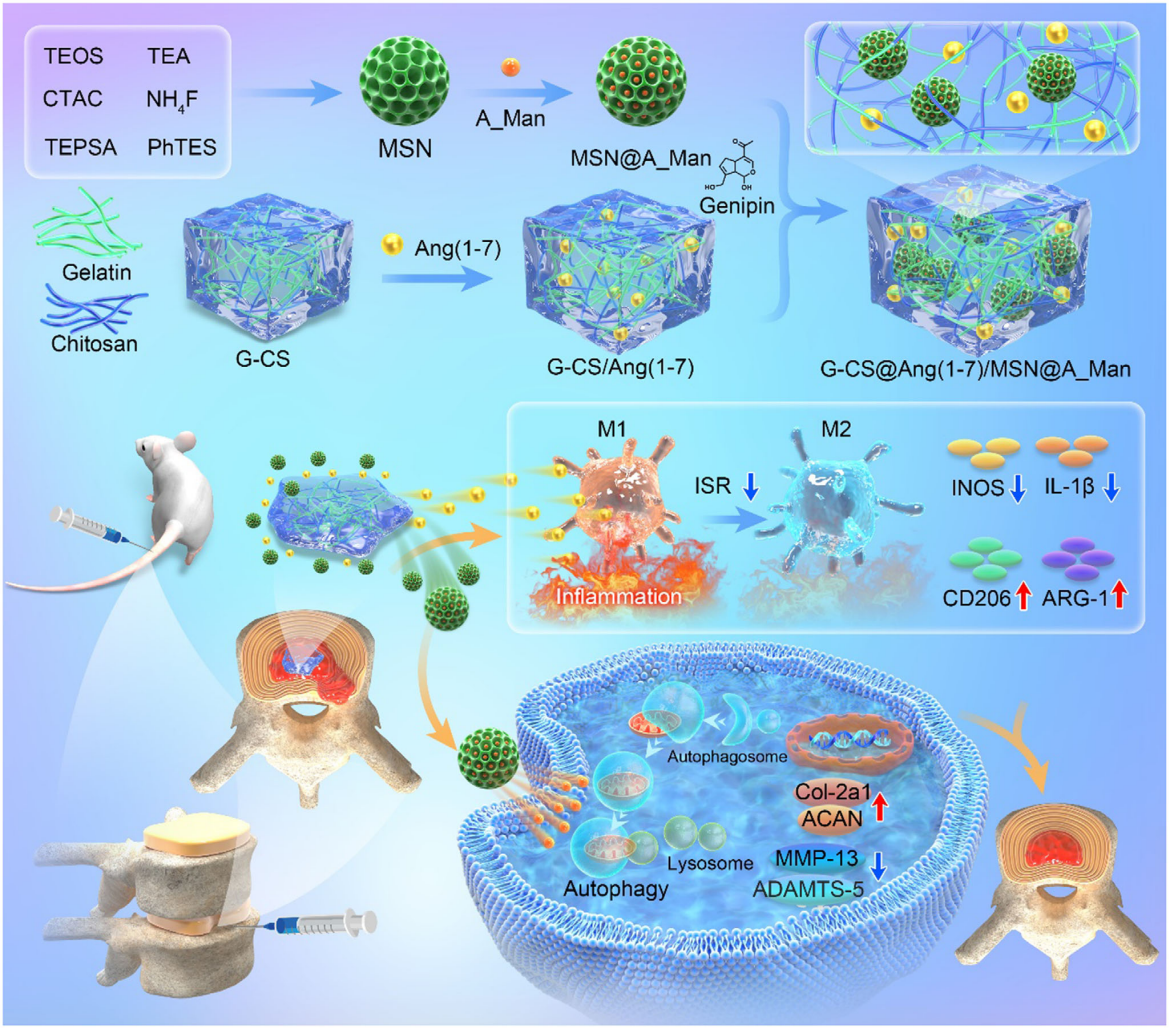
Design and schematic illustration of the G‐CS/MSN drug delivery system for IDD repair. A nanosphere hydrogel system capable of sequentially releasing two drugs was developed to achieve minimally invasive intradiscal injection therapy. This system can regulate macrophage polarization to improve the inflammatory environment, alleviate ECM metabolic disorders, and enhance autophagy in NPCs.

In this study, we design a novel material that is gelatin–chitosan (G‐CS) hydrogels encapsulating MSN. We incorporate Ang (1‐7) and A_Man into the materials to develop a G‐CS@Ang (1‐7)/MSN@A_Man (G‐CS/MSN) delivery system, which exploits the solubility differences between Ang (1‐7) and A_Man (with Ang (1‐7) being water‐soluble and A_Man being lipid‐soluble).^[10b]^ Our results demonstrate that the G‐CS/MSN effectively facilitates the regeneration and repair of NP (**Scheme** **1**).

## 2 Results

### 2.1 Preparation and Characterization of G‐CS/MSN

**Figure 1:**
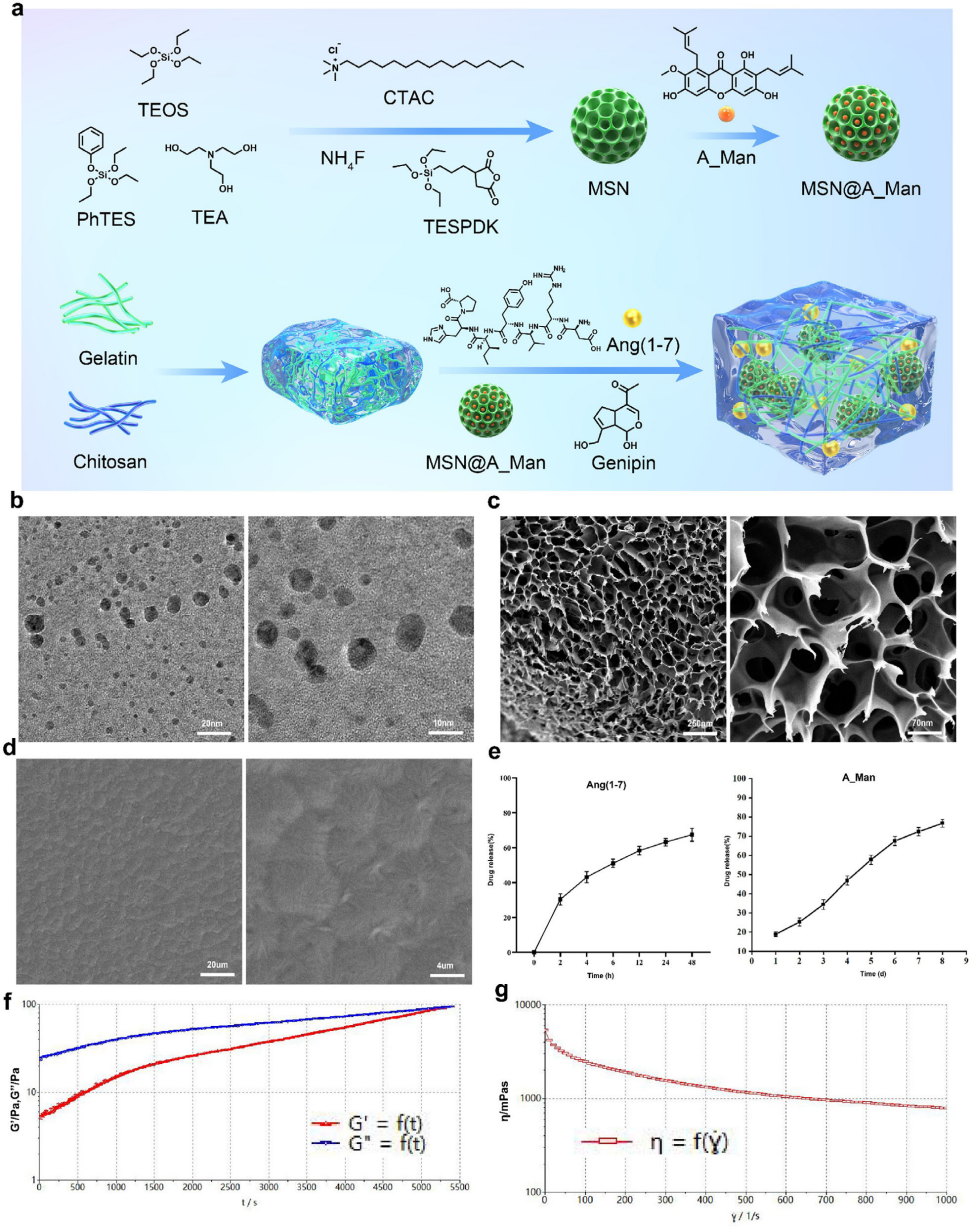
Preparation and characterization of G‐CS/MSN. a) Schematic diagram of the synthesis process of G‐CS/MSN. b) TEM detection of MSN (scale bar: 20nm, and 10 nm). c) SEM detection of MSN (scale bar: 250nm, and 70 nm). d) SEM detection of G‐CS (scale bar: 20 µm, and 4 µm). e) Release curves of two drugs in the G‐CS/MSN drug delivery system. f) Modulus test: storage modulus G′/loss modulus G″. g) The dynamic viscosity test shows the linear variation of the shear rate.

A two‐step process was used to prepare the G‐CS/MSNs (**Figure** **1**a). The hydrogel injected into the IVD must form strong adhesive bonds with both the annulus fibrosus and the vertebral endplates. This robust adhesion prevents hydrogel displacement or extrusion during spinal movement while ensuring uniform mechanical load distribution. Our tests demonstrate that the G‐CS hydrogel maintains excellent adhesion under mechanical stresses such as twisting and folding, both in ambient air and PBS (Figure S1a, Supporting Information). These results suggest that G‐CS can meet the demanding adhesive requirements within the complex biomechanical environment of the IVD. The hydrogel also has remarkable swelling resistance in PBS over varying periods (0, 6, 12, and 24 h, Figure S1b, Supporting Information). Meanwhile, Adhesion testing on porcine skin revealed that the hydrogel could withstand tensile stresses up to 6.5 kPa, confirming its robust mechanical properties for biomedical applications (Figure S1c, Supporting Information). Next, we characterized the G‐CS and MSN using electron microscopy to evaluate their microstructure and surface morphology. Transmission electron microscopy (TEM) was used to observe the morphology of the MSN (Figure 1b). Scanning electron microscopy (SEM) was used to observe the overall distribution of surface pores on the MSN (Figure 1c) and G‐CS (Figure 1d). MSN surface pores were evenly distributed, indicating good drug‐loading properties. The drug loading efficiency of MSN@A_Man and G‐CS@Ang(1‐7) is 51.11% and 80.76%. The SEM images of G‐CS degradation at 3 and 7 days demonstrate a surface erosion‐dominated degradation mechanism, as shown in Figure S2 (Supporting Information), indicating its potential for in vivo degradation and controlled drug release profiles. We next examined the drug release capacity of the G‐CS/MSN system. The release profiles of the two drugs in the G‐CS/MSN drug delivery system followed a sequential pattern (Figure 1e). The release curves demonstrated a biphasic release, with Ang (1‐7) showing an early burst and A_Man exhibiting more sustained release over time, weather in G‐CS/MSN or MSN@A_Man (Figure S3, Supporting Information). These curves are critical for ensuring effective treatment of IDD. We further conducted rheological testing on G‐CS/MSN. As the experiment progressed, both the storage and loss moduli of G‐CS/MSN gradually increased until the gel point was reached (Figure 1f). The dynamic viscosity test showed that G‐CS/MSN exhibited significant shear‐thinning characteristics, demonstrating its potential for effective injection into the IVD (Figure 1g).

### 2.2 G‐CS/MSN Exhibits Excellent Biocompatibility

**Figure 2:**
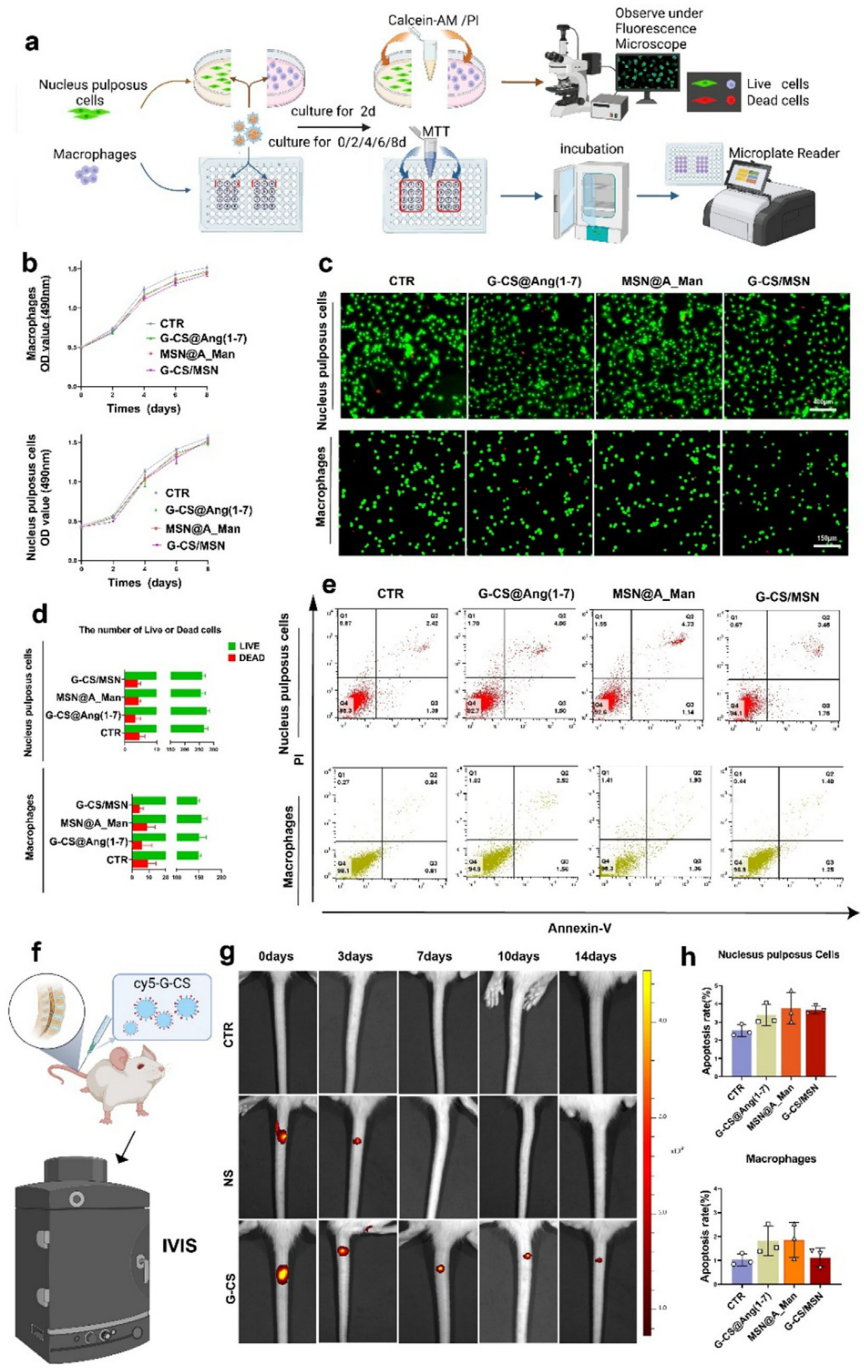
Biocompatibility of G‐CS@Ang (1‐7), MSN@A_Man, and G‐CS/MSN and degradation of G‐CS hydrogel. a) Experimental procedure for MTT assays and live/dead staining (created using BioRender.com). b) MTT assay results for rat NPCs and macrophages after different treatments (n = 3). c) Live/dead staining of rat NPCs and macrophages following different interventions (n = 3, scale bar: 400, and 150 µm). d) Quantitative analysis of live/dead staining images, providing data on the percentage of live and dead cells. e) Flow cytometry assays detecting apoptosis in rat NPCs and macrophages after different treatments (n = 3). f) Experimental procedure for IVIS (created using BioRender.com). g) IVIS images showing the distribution of CY5‐labeled G‐CS hydrogel in the intervertebral disc at different time points: includes images for CTR (negative control, no injection) and NS (positive control, injection of CY5‐labeled saline) (n = 3). h) Percentage of apoptotic NPCs and macrophages after different treatments.

Excellent biocompatibility of biomaterials is crucial to their effective functioning. To confirm biocompatibility, we performed MTT assays and cell live/dead staining to evaluate the effects of biomaterials on cell proliferation and cytotoxicity (**Figure** **2**a). The results showed no significant differences in the results of MTT for either macrophages or NPCs when treated with G‐CS@Ang (1‐7), MSN@A_Man, or G‐CS/MSN at various time points (Figure 2b). To further assess whether these materials exhibit cytotoxicity, we performed live/dead cell staining (Figure 2c). Statistical analysis revealed no significant differences in the proportion of live versus dead cells between the groups treated with the three materials and the control group (Figure 2d). Additionally, apoptosis was detected by flow cytometry, and the results showed that the apoptosis rates of macrophages and NPCs were less than 3% and 5%, respectively (Figure 2e,h). These results demonstrate that G‐CS@Ang (1‐7), MSN@A_Man, and G‐CS/MSN exhibited excellent biocompatibility in vitro, which forms the basis for subsequent experiments. We then extracted and examined tissue sections from various rat organs, including the heart, liver, spleen, lungs, and kidneys. Hematoxylin and eosin (H&E) staining revealed an orderly structure of organ sections and regular cellular morphology, with no significant signs of toxicity (Figure S4, Supporting Information). Finally, to investigate the degradation of the G‐CS hydrogel in rats, we injected CY5‐labeled G‐CS into the IVD. The hydrogel was monitored using an in vivo animal imaging system, with rats injected with CY5‐labeled normal saline and non‐injected rats serving as controls (Figure 2f,g). The results showed that G‐CS degraded rapidly within three days after injection into the IVD, indicating prime biodegradability and drug release ability.

### 2.3 G‐CS@Ang (1‐7) Alleviates LPS‐Induced M1 Polarization of Rat Macrophages by Inhibiting the Integrated Stress Response and Promoting the Polarization of M0 Macrophages Toward M2

**Figure 3:**
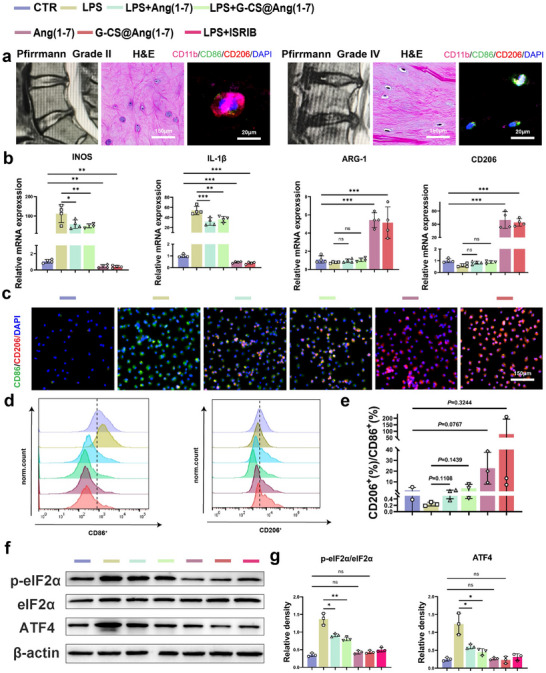
Induction of macrophage polarization by G‐CS@Ang (1‐7). a) MRI images, H&E, and IF for macrophage surface markers (CD11b, CD206, CD86) in NPCs from individuals with different grades of disc degeneration (n = 5, scale bar: 150 µm, and 20 µm). b) qPCR results showed the expression levels of INOS, IL‐1β, ARG‐1, and CD206 RNA in macrophages after different treatments (n = 4). c) IF of macrophage surface markers (CD206, CD86) after different interventions (n = 3, scale bar: 150 µm). d) Flow cytometry results quantifying the number of CD86^+^ and CD206^+^ macrophages following different treatments (n = 3). e) Statistical analysis of the proportion of CD206^+^ and CD86^+^ macrophages. f) Western blot results for different treatment groups of eIF2α, p‐eIf2α, and ATF4. g) Statistical analysis of western blot results (*P < 0.05, **P < 0.01, ***P < 0.001).

To determine the correlation between IDD and macrophages, we collected NP derived from individuals with varying degrees of degeneration. The NP was sectioned and subjected to H&E and immunofluorescence (IF) staining (**Figure** **3**a). Compared with Pfirrmann grade II, NP in Pfirrmann grade IV showed a higher number of M1 macrophages. This finding is consistent with those of previous studies, which showed that macrophages accumulated in the NP as the grade of degeneration increased, with M1 macrophages constituting the dominant portion.^[^ ^17^ ^]^ Therefore, we wanted to reverse macrophage polarization to alleviate IDD. Next, we evaluated the effects of Ang (1‐7) and G‐CS@Ang (1‐7) on macrophage polarization. We used real‐time quantitative polymerase chain reaction (qPCR) to evaluate the expression level of M1/M2 polarization marker RNA in macrophages of different treatment groups (Figure 3b). For LPS‐stimulated macrophages, both Ang (1‐7) and G‐CS@Ang (1‐7) effectively reduced the expression of INOS and IL‐1β (*P* < 0.05) but did not significantly increase ARG‐1 and CD206 levels (*P* > 0.05). In the treatment of macrophages that are not induced to polarize, both Ang (1‐7) and G‐CS@Ang (1‐7) effectively reduced the expression of INOS and IL‐1β and significantly increased the expression of ARG‐1 and CD206 (*P* < 0.05). Next, we used IF (Figure 3c) and flow cytometry (Figure 3d) to further assess macrophage polarization. The polarization status was analyzed by comparing the ratio of CD206^+^ to CD86^+^ cells (Figure 3e; Figure S5, Supporting Information). Although there is no statistically significant difference in the data, a clear trend of change can be observed. Ang (1‐7) and G‐CS@Ang (1‐7) effectively reduced the number of CD86^+^ macrophages following LPS stimulation (*P* < 0.05) and increased the number of CD206^+^ cells in the unstimulated group. Collectively, these results indicate that Ang (1‐7) and G‐CS@Ang (1‐7) can effectively reduce the extent of M1 polarization in macrophages and promote the polarization of M0 macrophages toward the M2 phenotype.

Finally, we investigated the anti‐inflammatory mechanisms of Ang (1‐7). A key marker of integrated stress response (ISR) activation is eIF2α phosphorylation at the Ser‐51 site.^[^ ^18^ ^]^ Western blotting was performed to analyze the expression of this protein in macrophages using a small‐molecule ISR inhibitor, ISRIB.^[^ ^19^ ^]^ After LPS treatment, the levels of ATF4 and p‐eIF2α/eIF2α were elevated in macrophages. However, Ang (1‐7) and G‐CS@Ang (1‐7) effectively reduced the expression of these proteins (Figure 3f,g). This indicates that Ang (1‐7) can mediate ISR to inhibit LPS‐induced proinflammatory polarization in macrophages.

### 2.4 MSN@A_Man Effectively Inhibits Inflammation, Improves ECM Metabolism Dysregulation, and Alleviates NPC Aging and Death

**Figure 4:**
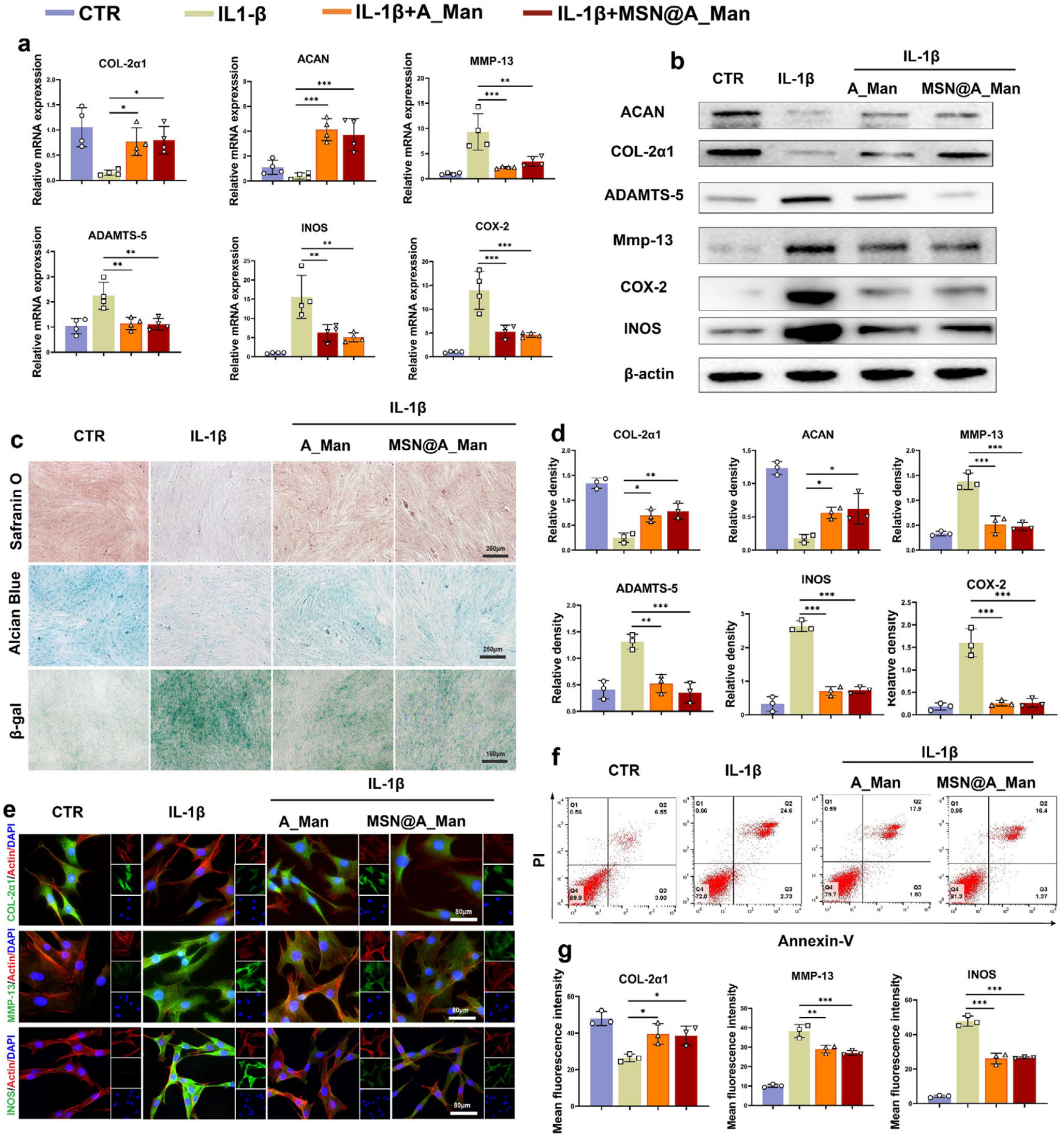
MSN@A_Man maintains ECM metabolism balance and alleviates inflammation in NPCs. a) qPCR results showing the expression of ECM synthesis markers (COL‐2α1, ACAN), degradation markers (ADAMTS‐5, MMP‐13), and inflammation indicators (COX‐2, INOS) after different treatments (n = 4). b) Western blot results for ECM synthesis proteins (COL‐2α1, ACAN), degradation proteins (ADAMTS‐5, MMP‐13), and inflammation markers (COX‐2, INOS) following different interventions (n = 3). c) Safranin O, Alcian Blue, and β‐gal staining of NPCs after various treatments (n = 3, scale bar: 250 µm, and 150 µm). d) Quantitative analysis of western blot data. e) IF of COL‐2α1, MMP‐13, and INOS in NPCs after different treatments (n = 3, scale bar: 80 µm). f) Flow cytometry results assessing the apoptosis of NPCs following different interventions (n = 3). g) Statistical analysis of fluorescence intensity from IF analysis (*P < 0.05, **P < 0.01, ***P < 0.001).

ECM is a crucial component of NP, broadly categorized into ECM synthesis and degradation. An inflammatory environment in the degenerated NP can lead to an imbalance between ECM synthesis and degradation.^[^ ^20^ ^]^ To verify that A_Man and MSN@A_Man can effectively counteract inflammatory factors and improve the microenvironment, we used IL‐1β to mimic the inflammatory environment in vivo. QPCR was used (**Figure** **4**a) to assess the RNA expression of genes related to ECM synthesis (Col‐2α1, ACAN), degradation (ADAMTS‐5, MMP‐13), and inflammation (INOS, COX‐2). Western blotting (Figure 4b,d) and IF (Figure 4e,g) were used to assess the protein levels of these indicators. The results showed that both A_Man and MSN@A_Man improved the reduced expression of ECM synthesis markers and counteracted the increased expression of degradation and inflammation markers induced by IL‐1β (*P* < 0.05). We further stained the NPCs with Safranin O and Alcian Blue (Figure 4c). The intensity of staining reflects the level of collagen synthesis. The results indicated that A_Man and MSN@A_Man can effectively mitigate the decrease in collagen synthesis induced by IL‐1β. β‐gal is often used to detect senescent cells.^[^ ^21^ ^]^ We found that A_Man and MSN@A_Man simultaneously ameliorated cell senescence induced by IL‐1β (Figure 4c). Finally, flow cytometry was performed to assess apoptosis (Figure 4f; Figure S6, Supporting Information). The results indicated that A_Man and MSN@A_Man effectively improved cell apoptosis induced by IL‐1β. The above experiments demonstrated that A_Man and MSN@A_Man have excellent anti‐inflammatory effects and can effectively improve the dysregulation of ECM metabolism caused by inflammatory stimuli, alleviating NPC aging and death.

### 2.5 MSN@A_Man Inhibits Inflammation‐Mediated Activation of the PI3K/AKT/mTOR Signaling Pathway and Promotes the Ubiquitin‐Dependent Pink1/Parkin Pathway, Enhancing Mitophagy

**Figure 5:**
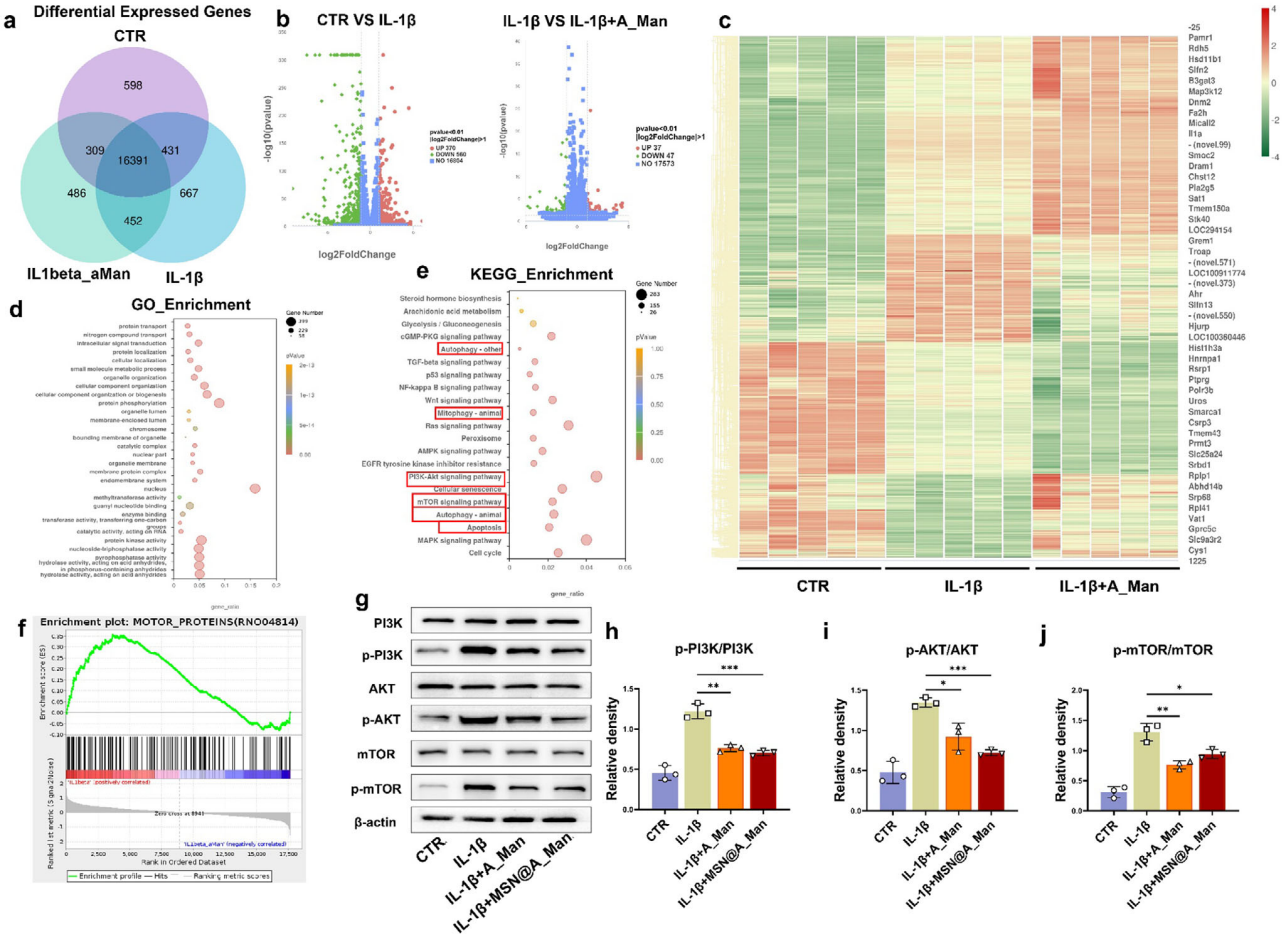
MSN@A_Man antagonizes IL‐1β‐induced cellular pathways. a) Venn diagram showing the overlap of differentially expressed genes across different treatment groups. b) Volcano plot illustrates the significance and fold‐change of differentially expressed genes between treatment groups. c) Heatmap showing the expression levels of differentially expressed genes across various treatment conditions. d) GO enrichment analysis of differentially expressed genes between IL‐1β and IL‐1β+A_Man groups. e) KEGG pathway enrichment analysis for differential genes between IL‐1β and IL‐1β+A_Man groups. f) GSEA showing enrichment of differential genes in the mTOR pathway between IL‐1β and IL‐1β+A_Man groups. g) Western blot detection of key proteins in the PI3K/AKT/mTOR pathway, including p‐PI3K, PI3K, p‐AKT, AKT, p‐mTOR, and mTOR, in different treatment groups (n = 3). h–j) Quantitative analysis of western blot data for proteins in the PI3K/AKT/mTOR pathway (*P < 0.05, **P < 0.01, ***P < 0.001).

We performed RNA sequencing to understand the molecular mechanisms by which MSN@A_Man alleviates ECM metabolic disorders. The Venn diagram showed that 16391 genes were co‐expressed in the three groups of cells (**Figure** **5**a). Differential gene analysis was then conducted. We performed pairwise comparisons between the CTR, IL‐1β, and IL‐1β+A_Man groups. Volcano plots revealed 370 upregulated genes and 560 downregulated genes in the control versus IL‐1β comparison and 37 upregulated genes and 47 downregulated genes in the IL‐1β versus IL‐1β+A_Man comparison, with |log2FC| ≥ 1 and P ≤ 0.01 (Figure 5b). The cluster heat map showed genes associated with changes in NPCs and depicted multiple changes in gene expression across the three groups (Figure 5c). These results indicated a broad range of differences in gene expression. Next, we performed Gene Ontology enrichment analysis on the differentially expressed genes between IL‐1β and IL‐1β+A_Man groups (Figure 5d). The results showed that A_Man improved various aspects of NPCs biology, including biological processes, cellular components, and molecular functions following inflammatory stimulation. Finally, we conducted Kyoto Encyclopedia of Genes and Genomes (KEGG) enrichment (Figure 5e) and Gene Set Enrichment Analysis (GSEA) enrichment (Figure 5f) analyses on the differentially expressed genes between the IL‐1β and IL‐1β+A_Man groups. These results revealed that A_Man had a significant regulatory effect on the PI3K/AKT/mTOR pathway. Previous studies have shown that IL‐1β can activate the PI3K/AKT/mTOR pathway, which inhibits chondrocyte proliferation and promotes chondrocyte apoptosis.^[^ ^22^ ^]^ We also detected key proteins in the PI3K/AKT/mTOR pathway using western blotting (Figure 5g–j). The results showed that both A_Man and MSN@A_Man inhibited activation of the PI3K/AKT/mTOR pathway by reducing the phosphorylation of PI3K, AKT, and mTOR proteins (P < 0.05).

**Figure 6:**
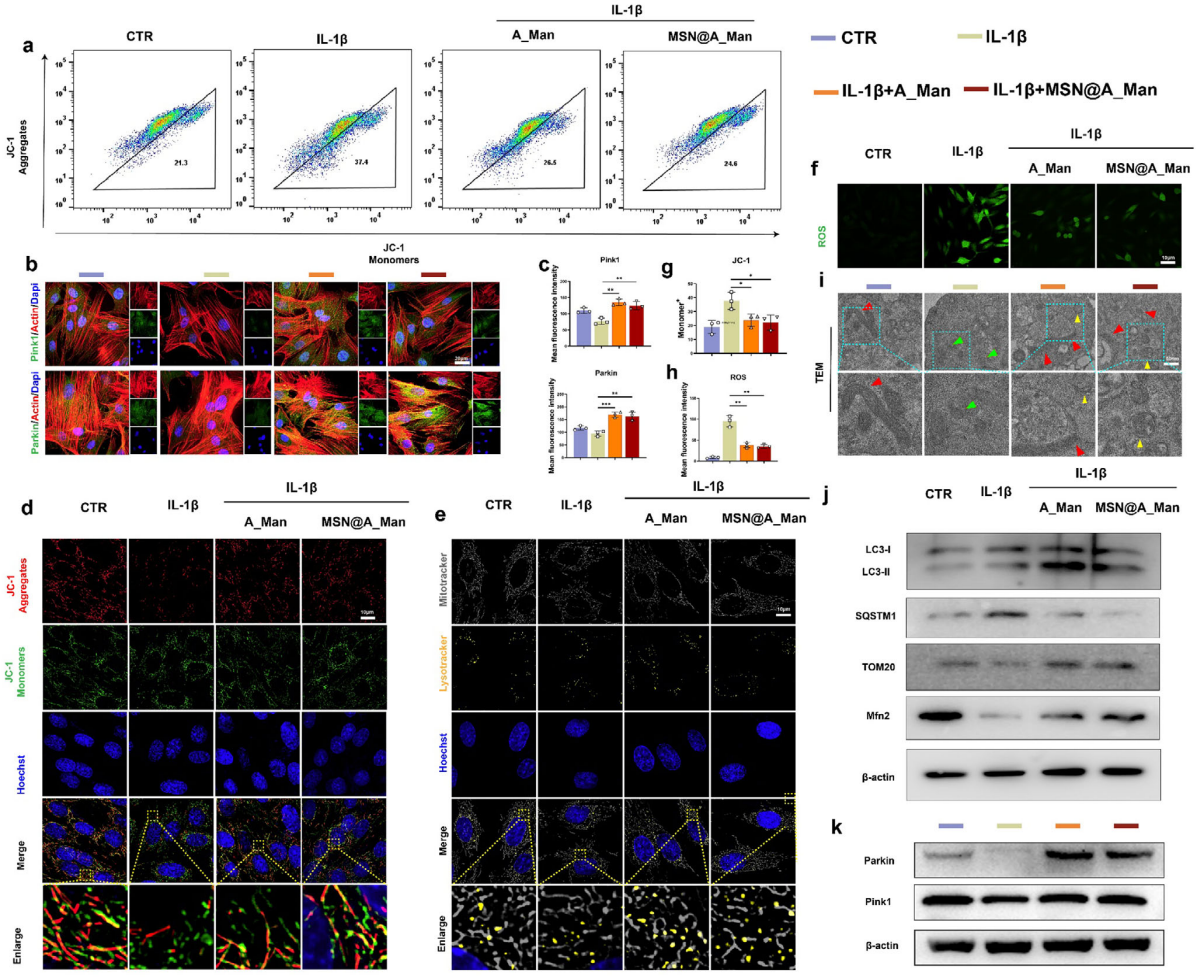
MSN@A_Man enhances mitochondrial autophagy through the Pink1/Parkin pathway in NPCs. a) Flow cytometry analysis of JC‐1 staining to assess mitochondrial membrane potential changes under different treatments (n = 3). b) IF of key proteins involved in mitochondrial autophagy (Parkin and Pink1) under different interventions (n = 3, scale bar: 20, and 150 µm). c) Statistical analysis of the fluorescence intensity of Parkin and Pink1 from IF. d) IF staining images of JC‐1 under various treatment conditions (n = 3, scale bar: 10 µm). e) Co‐localization IF staining of MitoTracker and LysoTracker to observe mitochondrial–lysosome interactions under different treatments (n = 3, scale bar: 10 µm). f) IF showing ROS levels under different treatment conditions (n = 3, scale bar: 10 µm). g) Quantitative analysis of JC‐1 flow cytometry data. h) Statistical analysis of ROS fluorescence intensity. i) TEM images showing mitochondrial morphology and structure; red arrows indicate normal mitochondria with clear cristae, green arrows indicate mitochondrial shrinkage and pigmentation, and yellow arrows indicate autophagic lysosomes (n = 3, scale bar: 50 nm). j) Western blot detection of LC3, SQSTM1, TOM20, and Mfn2 expression levels in nucleus cells under different treatments (n = 3) k) Western blot detection of Parkin and Pink1 expression levels in nucleus cells under different treatments (n = 3) (*P < 0.05, **P < 0.01, ***P < 0.001).

Autophagy is a conserved cellular metabolic pathway that maintains intracellular stability, plays a crucial regulatory role in inflammation, and affects the pathological progression of inflammatory diseases.^[^ ^23^ ^]^ The PI3K/AKT/mTOR pathway is widely recognized as a fundamental intracellular signaling pathway involved in normal cell physiology and cancer pathology that inhibits autophagy when activated.^[^ ^24^ ^]^ We previously demonstrated that A_Man and MSN@A_Man effectively inhibit the PI3K/AKT/mTOR pathway. KEGG enrichment (Figure 5e) analyses also indicated that A_Man has a significant impact on regulating autophagy in NPCs. Flow cytometry was used for JC‐1 analysis (**Figure** **6**a) to verify the protective effect on cells under inflammation. After IL‐1β stimulation, the proportion of mitochondrial aggregates decreased, whereas the proportion of monomers increased, with CCCP^+^ serving as a positive control (Figure S7, Supporting information). Both A_Man and MSN@A_Man effectively reduced the proportion of monomers in the NPCs (P < 0.05) (Figure 6g). According to the IF of JC‐1, the fluorescence intensity of mitochondrial aggregates decreased after IL‐1β stimulation, whereas that of monomers increased, indicating severe mitochondrial fragmentation. The fluorescence of aggregates was significantly enhanced from that in the IL‐1β group, and the mitochondrial morphology appeared more regular and intact after the addition of A_Man or MSN@A_Man (Figure 6d; Figure S8, Supporting information). This confirmed the protective effect of A_Man or MSN@A_Man on mitochondria. Our subsequent investigation into mitochondrial and autophagy‐related proteins revealed that both A_Man and MSN@A_Man effectively enhanced the conversion of LC3‐I to LC3‐II while suppressing SQSTM1/p62 expression, thereby ameliorating mitophagy activation. Furthermore, both A_Man and MSN@A_Man can antagonize IL‐1β‐induced mitochondrial damage, as evidenced by elevated expression levels of Mfn‐2 and TOM20 in NPCs (Figure 6j; Figure S9, Supporting Information). Next, we investigated the expression of key pathway proteins (Parkin, Pink1) in mitophagy in NPCs using western blotting and IF (Figure 6b,c,k; Figure S9, Supporting Information). The results showed that IL‐1β suppressed the expression of the aforementioned proteins, whereas A_Man or MSN@A_Man counteracted the effects of IL‐1β, leading to increased expression of Parkin and Pink1 proteins (P < 0.05). Co‐localization fluorescence staining was performed with MitoTracker and LysoTracker to further verify the changes of mitophagy. Both A_Man and MSN@A_Man significantly increased the co‐localization area (P < 0.05) (Figure 6e; Figure S10, Supporting information). To observe the phenomenon of mitophagy in the NPCs more clearly, we conducted TEM to observe the mitochondrial structure (Figure 6i). Mitochondria in NPCs showed significant shrinkage, loss of cristae, and prominent pigment deposition (green arrows) after IL‐1β stimulation compared to the control group (red arrows: normal mitochondria). In contrast, A_Man and MSN@A_Man groups exhibited restored mitochondrial morphology with a higher number of autophagolysosomes (yellow arrows). ROS staining was also performed (Figure 6f,h), which showed that the addition of A_Man or MSN@A_Man effectively reduced the intracellular ROS content from that in the IL‐1β group (P < 0.05). The above experiments collectively demonstrate that MSN@A_Man can improve inflammation‐mediated mitochondrial structural and functional damage and enhance mitophagy.

### 2.6 G‐CS/MSN Counteracts Inflammatory Stimuli, Improves ECM Metabolic Disorders, Alleviates Cellular Aging, and Reduces Cell Death

**Figure 7:**
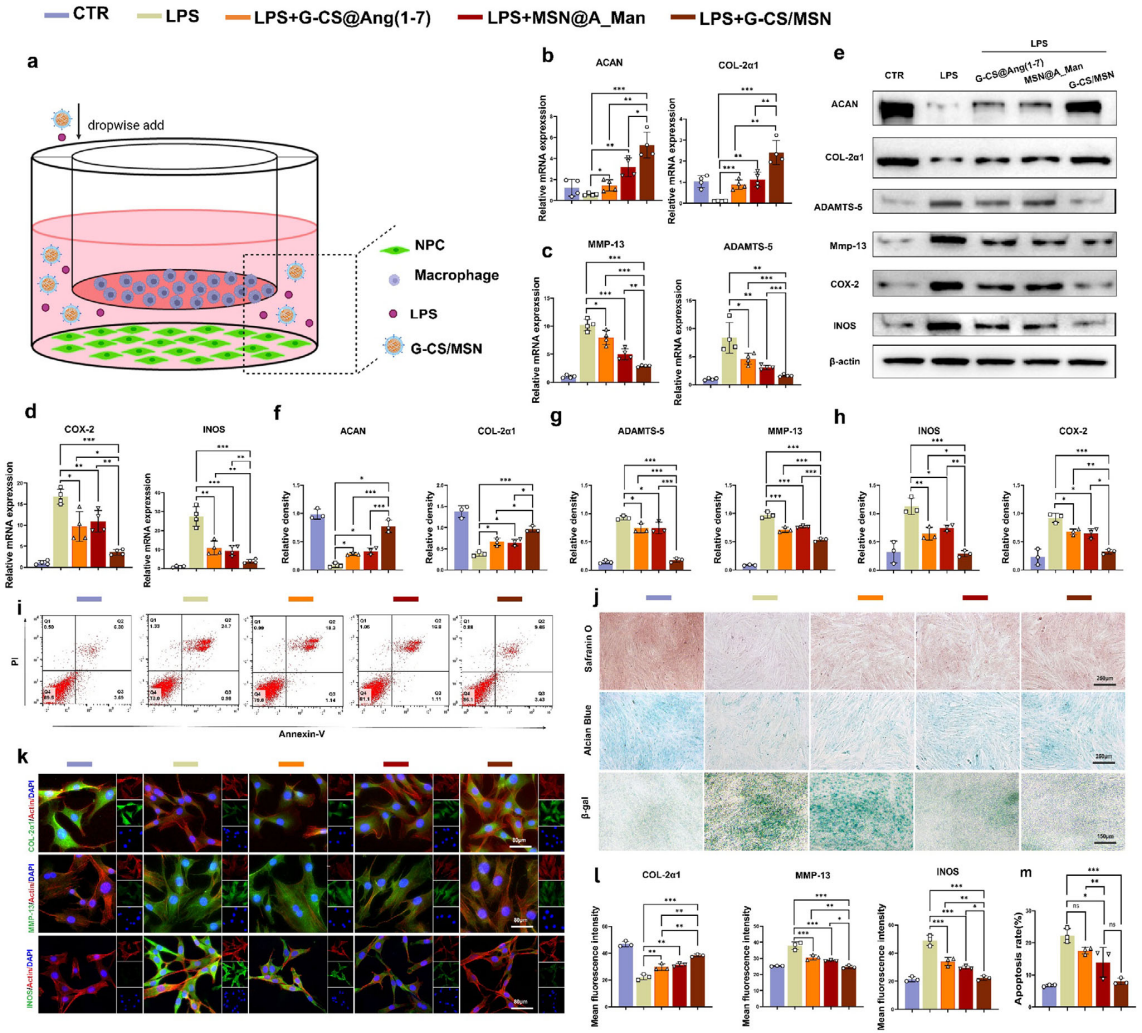
G‐CS/MSN effectively counteracts inflammatory stimuli and maintains ECM metabolic balance in the macrophage/NPC co‐culture system. a) Co‐culture system setup (created using BioRender.com). b–d) qPCR analysis of the RNA expression of NPCs. e) Western blotting to assess protein expression levels of NPCs. f–h). Quantitative analysis of Western blot results. i). Flow cytometry analysis of apoptosis in NPCs under different interventions (n = 3). j) Safranin O, Alcian Blue, and β‐gal staining of NPCs under various treatments (n = 3, scale bar: 250 µm, and 150 µm). k) Statistical analysis of fluorescence intensity from IF (n = 3, scale bar: 80 µm). l) Quantitative analysis of IF. m) Quantitative analysis of flow cytometry results for apoptosis (*P < 0.05, **P < 0.01, ***P < 0.001).

Previously, we demonstrated the functions of these biomaterials. We innovatively used nanomaterials engineering to combine the two drugs to achieve the sequential release of the two medications for anti‐inflammation and repair IVD. The effect of the combined G‐CS@Ang (1‐7)/MSN@A_man (G‐CS/MSN) on cells was verified. We used a co‐culture system with macrophages in the upper chamber and NPCs in the lower chamber to simulate in vivo intercellular interactions (**Figure** **7**a). QPCR results showed that G‐CS@Ang (1‐7), MSN@A_Man, and G‐CS/MSN effectively counteracted inflammatory stimuli, enhanced ECM synthesis (Figure 7b), reduced degradation(Figure 7c), and alleviated inflammation (INOS, COX‐2) (Figure 7d). G‐CS/MSN demonstrated superior effectiveness to the other two groups (*P* < 0.05). Western blot analysis of protein expression levels (Figure 7e–h) confirmed the PCR results. Apoptosis was assessed by flow cytometry. The results showed that G‐CS@Ang (1‐7), MSN@A_Man, and G‐CS/MSN effectively improved IL‐1β‐induced cell apoptosis, with G‐CS/MSN demonstrating the most pronounced effect (Figure 7i,m). We also performed Safranin O and Alcian Blue staining to assess collagen synthesis, and β‐gal staining to evaluate cellular senescence (Figure 7j). Consistent with previous results, G‐CS/MSN significantly enhanced collagen synthesis and alleviated inflammation‐induced cell senescence. Finally, we used IF staining to further examine the protein expression of COL‐2α1, MMP‐13, and INOS in NPCs and compared the fluorescence intensity of these markers across the different groups. The results showed that G‐CS/MSN was more effective than the other two groups in enhancing COL‐2α1 expression and reducing MMP‐13 and INOS expression (Figure 7k,l).

These results indicate that our designed G‐CS@Ang (1‐7)/MSN@A_Man system, through sequential drug release, effectively exhibited anti‐inflammatory properties, improved ECM metabolic disorders induced by inflammation, inhibited cell apoptosis, and regulated cell aging. The next step involved conducting in vivo experiments to validate the efficacy of this material in repairing IDD.

### 2.7 G‐CS/MSN Effectively Promotes IVD Repair In Vivo

**Figure 8:**
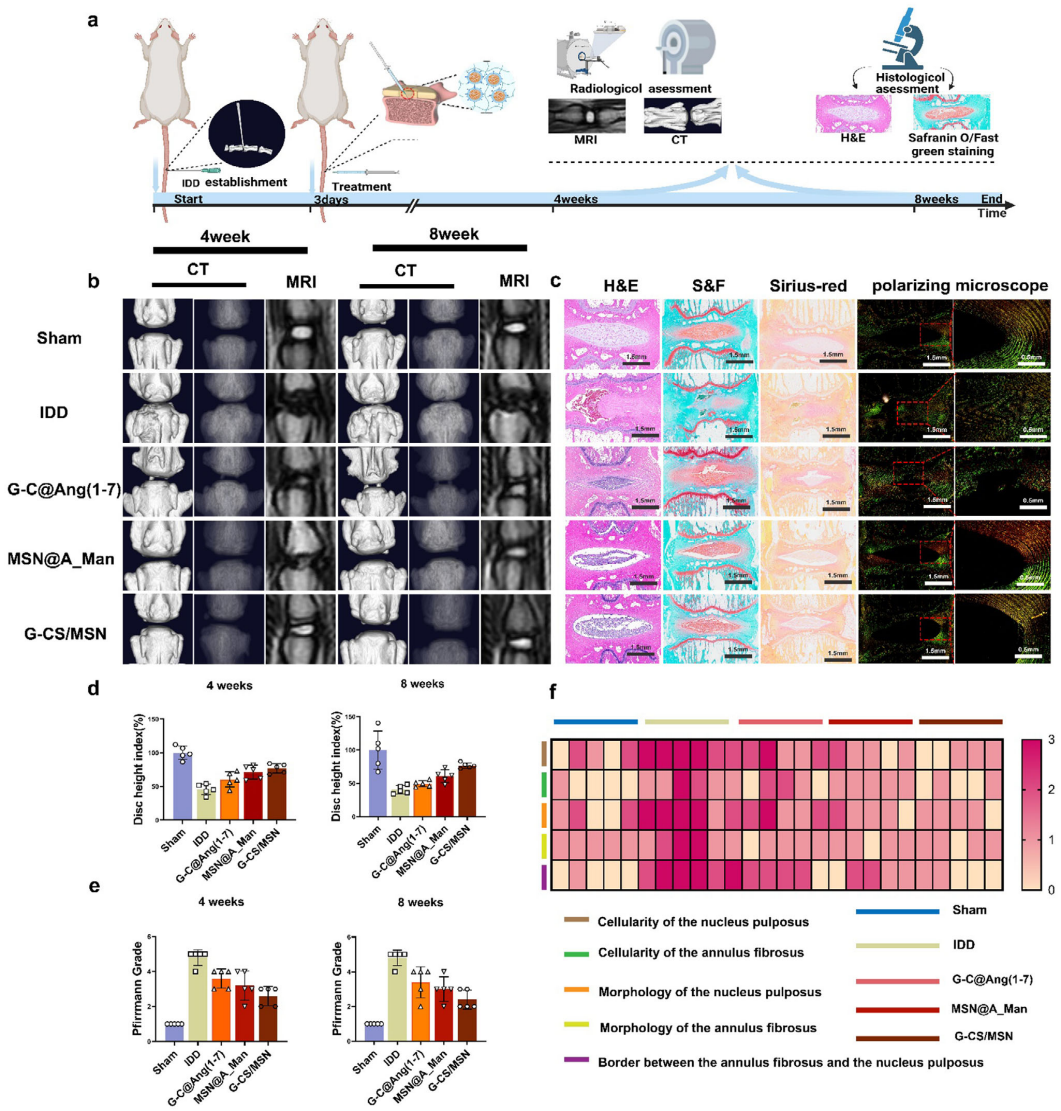
In vivo imaging results and evaluation of G‐CS/MSN in rats. a) Procedural flow for in vivo experiments in rats (created using BioRender.com). b) CT and MRI images of the punctured disc segments four and eight weeks after treatment (n = 5). c) H&E, Safranin O/Fast Green, Sirius Red, and polarizing microscope images of disc sections after eight weeks (n = 5, scale bar: 1.5 mm, 0.5 mm). d) Quantitative analysis of DHI at different time points for each treatment group. e) Quantitative assessment of the disc degeneration grade at different time points for each treatment group. f) Heatmap showing changes in histological scores across different treatment groups (*P < 0.05, **P < 0.01, ***P < 0.001).

We used a rat tail vertebral puncture model to verify whether G‐CS/MSN can promote repair and regeneration of the IVD. The rats were treated according to different groups, and computed tomography (CT) and magnetic resonance imaging (MRI) scans were performed at four and eight weeks. We calculated the disc height index (DHI) (Figure S11, Supporting information) and assessed the IDD grade.^[^ ^25^ ^]^ We also collected IVD segments from the model, performed histological sectioning at eight weeks (**Figure** **8**a), and then conducted histological scoring for different regions. CT revealed a significant reduction in disc height in the IDD group. In contrast, the G‐CS@Ang (1‐7) and MSN@A_man groups exhibited varying degrees of disc height recovery. Notably, the G‐CS/MSN group demonstrated superior disc height restoration compared with the other two groups at both four and eight weeks (Figure 8b,d). MRI‐T2WI was used to observe the degree of NPC degeneration (Figure 8b,e). The results showed varying degrees of recovery in the NP in G‐CS@Ang (1‐7), MSN@A_man, and G‐CS/MSN groups. However, the G‐CS/MSN group exhibited a significantly lower level of IVD degeneration than the other two groups.

We further examined the histology of NP, annulus fibrosus, and collagen expression in the IVD using various techniques, including H&E staining, Safranin O/Fast Green staining, Sirius Red staining, and polarized microscopy (Figure 8c). We also calculated histological scores to assess tissue repair and regeneration (Figure 8f). The sham group exhibited a clear disc structure with well‐rounded NP, orderly internal arrangement, and well‐defined boundaries. The annulus fibrosus was evenly distributed around NP with natural and clear boundaries. Safranin O/fast green staining revealed abundant red‐stained proteoglycans in NP, whereas the annulus fibrosus and bone displayed high levels of blue‐stained collagen. Sirius Red staining, combined with polarized microscopy, showed that the annulus fibrosus predominantly contained collagen types COL‐1 (yellow) and COL‐3 (green), whereas NP primarily consisted of COL‐2, which did not show significant color under polarized microscopy. In the IDD group, IVD showed significant degeneration, with most of the NP area replaced by fibrous structures. The internal structure of the NP was disorganized, and the boundaries were unclear. The IVD in the G‐CS@Ang (1‐7), MSN@A_Man, and G‐CS/MSN groups showed varying degrees of recovery. However, the G‐CS/MSN group demonstrated superior results to the other two groups in terms of NP morphology, area, annulus fibrosus morphology, and boundary clarity. This indicated that G‐CS/MSN was more effective in promoting IVD regeneration and repair in vivo.

**Figure 9:**
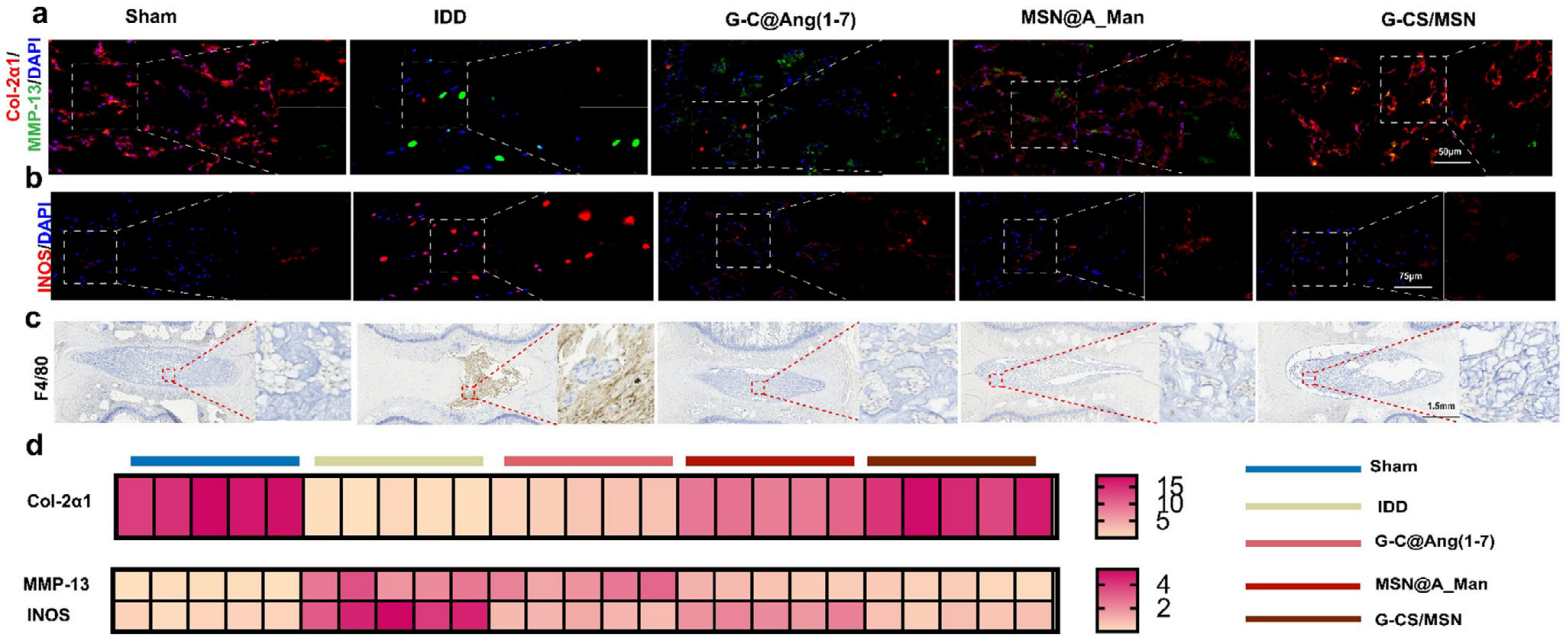
Histopathological analysis of rat nucleus pulposus tissue. a) IF of COL‐2α1 and MMP‐13 in different treatment groups (n = 5, scale bar: 50 µm). b) IF of INOS in different treatment groups (n = 5, scale bar: 75 µm). c) Immunohistochemical staining images of F4/80 in different treatment groups (n = 5, scale bar: 1.5 mm). d) Heatmap showing the fluorescence intensity of different indicators across the treatment groups.

Subsequently, the tissue sections were stained to assess relevant indicators. The IDD group showed reduced COL‐2α1 expression and increased levels of MMP‐13 and INOS in IVD. In contrast, the G‐CS@Ang (1‐7), MSN@A_Man, and G‐CS/MSN groups all demonstrated increased COL‐2α1 expression and decreased MMP‐13 and INOS levels. Among these, the G‐CS/MSN exhibited the most effective regulation (**Figure** **9**a,b,d). The macrophage‐specific marker F4/80 was used for IHC staining of macrophages. The results revealed a significant presence of macrophages in the NPCs of the IDD group, whereas no noticeable macrophage aggregation was observed in the treatment groups.

In summary, G‐CS/MSN protects the integrity of the IVD by improving the inflammatory environment and ECM metabolic disorders, halting the progression of IDD, and promoting regeneration and repair of the IVD through sequential drug release.

## 3 Discussion

Chitosan, a naturally derived polysaccharide featuring a β‐1,4‐linked glucosamine structure analogous to hyaluronic acid, serves as a critical component in both the ECM and synovial fluid of articular cartilage. Owing to its exceptional biocompatibility, tunable biodegradability, and low immunogenicity, chitosan has been extensively utilized as a scaffold material in tissue engineering applications.^[^ ^26^ ^]^ The thermoresponsive properties of gelatin (exhibiting low‐temperature sol state and body temperature‐induced gelation) make it an ideal candidate for injectable biomaterials to fill irregular IVD cavities conformally.^[^ ^27^ ^]^ Through physical or chemical crosslinking (e.g., genipin), chitosan‐gelatin composite hydrogels can form highly hydrated 3D network structures capable of achieving compressive modulus comparable to native IVD. This approach simultaneously circumvents the stress‐shielding effect associated with excessive modulus in traditional synthetic biomaterials.^[^ ^28^ ^]^ MSN, compared with traditional carriers (e.g., liposomes or polymer microspheres), exhibits unique advantages in drug‐loading capacity, controlled release kinetics, and microenvironment responsiveness, offering a novel strategy for precision therapy of IDD.^[^ ^29^ ^]^ By combining the advantages of G‐CS and MSN with sequential drug loading, we developed a G‐CS/MSN dual‐delivery system, providing a novel strategy to prevent IDD.

A multistage treatment strategy is more effective for repairing IDD, in which anti‐inflammatory drugs are followed by drugs that improve NPCs activity and function, providing a healthy environment for NPCs and enhancing treatment effectiveness.^[^ ^5^ ^]^ The phased treatment strategy is increasingly becoming the focus of disc regeneration therapy. We developed a G‐CS/MSN medicine delivery system designed to sequentially release therapeutic agents, improve the inflammatory environment within degenerated intervertebral discs, inhibit disc degeneration, and promote NPC regeneration. Additionally, we validated the potential of two drugs, Ang (1‐7) and A_Man, in intervertebral disc repair.In vivo, G‐CS initially degrades and releases Ang (1‐7), which modulates macrophage polarization and improves the inflammatory environment. Subsequently, the MSN encapsulated within the hydrogel is released, and enabling the sustained release of A_Man. This prolonged‐release corrects ECM metabolic imbalances in the NP, prevents cellular aging and death, promotes autophagy, and ultimately halts disc degeneration, promoting regeneration and repair of IDD.

Inflammation is the adaptive response of the body to mechanical, biochemical, or immune‐mediated stimuli. However, prolonged chronic inflammation within the IVD results in persistently elevated cytokine levels, which directly disrupt the balance of protein metabolism.^[^ ^30^ ^]^ This is also a contributing factor to inflammatory aging. The inflammatory process was initially thought to originate from macrophages, the primary cells of the innate immune system.^[^ ^31^ ^]^ Nevertheless, inflammation is now recognized as being induced and promoted by various age‐related processes,^[^ ^32^ ^]^ macrophage plasticity is a key factor in chronic inflammation.^[^ ^33^ ^]^ Attention should be paid to the influence of the microenvironment composed of macrophages on NPCs. In vivo, M1 macrophages secrete pro‐inflammatory cytokines such as TNF‐α, IL‐1β, IL‐6, and MCP‐1, whereas M2 macrophages secrete anti‐inflammatory and tissue repair‐promoting factors such as IL‐4, IL‐10, and TGF‐β.^[^ ^34^ ^]^ The disruption of this polarization balance is regarded as one of the key factors underlying the alteration of the IVD microenvironment. Regulating the microenvironment could control macrophage polarization, activation, and plasticity via complex gene networks and signaling cascades. Under inflammatory conditions, NPCs undergo significant metabolic alterations and may even experience abnormal cell death. In degenerated NPCs, the expression of p38 MAPK isoforms α, β, and δ is markedly activated. P38α and P38β play a critical role in inducing the release of GM‐CSF and IFNγ, which subsequently promote M1 polarization of macrophages, thereby exacerbating the inflammatory microenvironment in IDD.^[^ ^7a^ ^]^ Moreover, degenerated NPCs activate the MAPK‐ERK and PI3K‐AKT pathways, thereby upregulating the expression of MCP‐1 and MMP‐3. This process promotes M1 macrophage recruitment and exacerbates IDD.^[^ ^35^ ^]^ This dual‐targeting approach disrupts the self‐perpetuating loop of inflammation and tissue breakdown, offering a synergistic therapeutic advantage in preventing IDD. In our study, we demonstrated that Ang (1‐7) effectively promoted the polarization of M0 macrophages into M2 macrophages and counteracted LPS‐induced M1 polarization. Additionally, Ang (1‐7) alleviated the impact of the inflammatory environment on ECM metabolic dysregulation in co‐cultures of macrophages and NPCs. It holds significant potential as a therapeutic approach for the treatment of IDD. Through in vitro NPCs‐macrophage coculture systems and in vivo experiments, we demonstrated that Ang (1‐7) can effectively prevent the progression of IDD by modulating macrophage phenotypic switching. Further mechanistic investigations have also been conducted to elucidate this process. The ISR is a complex mechanism by which cells respond to various external pressures and stimuli. ISR activation regulates metabolic processes, protein synthesis, cell survival, and apoptosis when cells encounter adverse conditions, such as oxygen deprivation, nutrient deficiency, or DNA damage.^[^ ^36^ ^]^ Ang (1‐7) could counteract M1 macrophages polarization by inhibiting eIF2α phosphorylation and reducing ATF4 expression, suppressing the ISR. However, the detailed mechanisms require further investigation. Encapsulating Ang (1‐7) in G‐CS hydrogels allows for controlled drug release, which enhances the initial improvement in the inflammatory environment and establishes a strong foundation for disc repair.

The extracellular matrix (ECM) is a complex 3D network structure that comprises gel‐like molecules including collagen, elastin, integrin, and proteoglycans,^[^ ^37^ ^]^ which constitute the microenvironment and are closely linked to cell survival, regeneration, repair, and immune response.^[^ ^38^ ^]^ The gradual depletion of notochordal‐like and chondrocyte‐like nucleus pulposus (NPCs) leads to an imbalance in ECM metabolism during IDD. The proliferation of fibrochondrocyte‐like NPCs and excessive production of collagen type I frequently contribute to ECM metabolism dysregulation and the development of sclerosis.^[^ ^39^ ^]^ Therefore, protecting NPCs and maintaining ECM stability are crucial steps for improving IDD. In our designed G‐CS/MSN drug delivery system, A_Man was gradually released from MSN to address ECM metabolic disorders in NPCs. A_Man is a principal oxygenated anthraquinone purified from dietary plants,^[^ ^40^ ^]^ exhibiting excellent biocompatibility. We revealed that A_Man inhibits activation of the PI3K/AKT/mTOR pathway and enhances mitochondrial autophagy. The PI3K/AKT/mTOR pathway is a classic pathway in humans with substantial applications because of its potential roles in initiating inflammatory responses,^[^ ^41^ ^]^ regulating cell survival and apoptosis,^[^ ^42^ ^]^ and mediating autophagy.^[^ ^43^ ^]^ Mitochondrial dysfunction is a major factor contributing to the aging phenotype of NPCs.^[^ ^44^ ^]^ When mitochondrial function is disrupted by various conditions, such as high mechanical load and oxidative stress,^[^ ^45^ ^]^ damaged mitochondria must be promptly isolated and selectively removed.^[^ ^46^ ^]^ Therefore, mitophagy is essential for maintaining mitochondrial and cellular homeostasis.^[^ ^47^ ^]^ We found that A_Man effectively promoted mitophagy by activating the ubiquitin‐dependent Pink1/Parkin pathway. Meanwhile, A_Man improved mitochondrial membrane potential and reduced ROS expression in NP. RNA‐sequencing results indicated that A_Man is associated with TGF‐β activation, highlighting its significant potential in improving and repairing IVD. Loading A_Man onto MSN not only addressed the difficulty of delivery but also provided a controlled release.

This study has some limitations. Although we validated some of the mechanisms of action of these two drugs, we did not thoroughly investigate their downstream targets, which will be the focus of future work. Nevertheless, we demonstrated for the first time the significant efficacy of Ang (1‐7) and A_Man in treating IDD. Additionally, we designed a G‐CS/MSN sequential drug release system that boasts unique material properties and anti‐inflammatory and anti‐aging effects both in vitro and in vivo, providing a new approach to the treatment of IDD.

## 4 Conclusion

In this study, we propose an approach for the treatment of IDD, which exploits the different solubility properties of two drugs combined with bionanomaterials to create a sequential drug delivery system. In the first release phase, the outer layer of G‐CS releases Ang (1‐7), which suppresses ISR, regulates macrophage polarization, and improves the inflammatory environment within the disc. In the second phase, MSN releases A_Man, which improves ECM metabolism, and reduces cellular aging and apoptosis. Meanwhile, A_Man promotes mitophagy by inhibiting the PI3K/AKT/mTOR pathway and activating the ubiquitin‐dependent Pink1/Parkin pathway. This G‐CS/MSN drugs delivery system offers a novel direction for treating IDD.

## 5 Experimental Section

### Synthesis of MSN—Preparation of Polymer Solution

To obtain a polymer solution, a mixture of tetraethyl orthosilicate (TEOS, 1.62 g, 7.79 mmol) and phenyl triethoxysilane (0.45 g, 1.86 mmol) was added to triethanolamine (14.3 g, 95.6 mmol).

### Emulsification Process

The polymer solution was quickly added to a preheated solution of cetyltrimethylammonium chloride (0.586 g, 1.83 mmol) and ammonium fluoride (NH4F, 0.1 g, 2.7 mmol) in water (21.7 g, 1.21 mmol) at 60 °C, and stirred vigorously for 20 min while cooling to room temperature.

### Crosslinking Reaction

One quarter of TEOS (totaling 192.2 mg, 0.922 mmol) was added to the emulsion every 3 min, and the reaction mixture was stirred at room temperature for 30 min. Finally, TEOS (38.3 mg, 184 µmol) and 3‐(triethoxysilyl) propyl succinic anhydride (56.2 mg, 184 µmol) were added, and the resulting mixture was stirred at room temperature overnight.

### Recovery and Washing of Microspheres

The final reaction mixture was centrifuged (12 000 rcf, 20 min) to collect the MSN, which were then redispersed in anhydrous ethanol. The subsequent reaction of MSN was carried out in an ethanol solution (100 mL) containing 2 g of ammonium nitrate (NH4NO3), with refluxing at 90 °C for 45 min. After centrifugation (12 000 rcf, 15 min) and redispersion in ethanol, refluxing was repeated. Once the reaction mixture had cooled to room temperature, the MSN were collected by centrifugation (12 000 rcf, 15 min) and washed twice with 100 mL of anhydrous ethanol.

### Freeze‐Drying

The washed microspheres were placed in a freeze‐dryer to remove moisture, resulting in stable dry microspheres.

### Drug Loading

The MSNs (10 mg) were evenly dispersed in 5 mL of ultrapure water for later use. A total of 5 mg of A_Man was fully dissolved in 2 mL of methanol, which was then slowly dripped into ultrapure water containing the nanoparticles and stirred for 24 h. Methanol was allowed to evaporate slowly, and the resulting mixture was freeze‐dried to obtain MSN@A_Man.

### Preparation of G‐CS/MSN

Gelatin (40 mg) was completely dissolved in 2 mL of ultrapure water, followed by the addition of 100 mg of chitosan. The prepared drug‐loaded nanoparticles were added and stirred evenly. Finally, 200 µL of genipin solution (10 mg/mL) and 200 µL of Ang (1‐7) solution (10 mg/mL) were added, stirred thoroughly, and allowed to sit until gelation occurred, yielding G‐CS@Ang (1‐7)/MSN@A_Man.

### Physicochemical and Multifunctional Property Characterization of G‐CS/MSN

The MSN particle size was observed by TEM, and the surfaces of MSN and G‐CS were observed by SEM. Rheological properties, including the energy storage modulus (G′), loss modulus (G″), and shear rate, were determined using a rheometer (HAAKE MARS 40, Germany).

### Calculation of Drug Release Rate

Physiological saline or phosphate‐buffered saline (PBS) was chosen as the release medium, and the pH was adjusted to 7.4 to simulate physiological conditions. A certain amount of hydrogel microspheres was placed in a dialysis bag (MWCO = 1000), which was immersed in the release medium, maintaining a constant temperature of 37 °C while stirring at 100 r min^−1^ on a magnetic stirrer. At predetermined time points, 3.0 mL of the release medium was withdrawn, and an equal volume of PBS was added. The concentration of the released drug was analyzed by UV–vis spectrophotometry. All release measurements were conducted in triplicate, and the average values were plotted. The amount of drug released was compared to the initial amount of drug, and the release rate was calculated using the following Equation (1):

(1) $$E_{r}=\frac{V_{e}\sum_{1}^{n-1}C_{i}+V_{0}C_{n}}{m_{drug}}$$

where *Er* is the cumulative drug release; *Ve* is the volume of replaced PBS; *V0* is the total volume of release medium; *Ci* is the concentration of release medium during the ith sampling; *m_drug_* is the total drug mass; and *n* is the number of times PBS was replaced.

(2) $$Release\;Rate=\frac{Amount\;of\;Drug\;Released}{Initial\;Amount\;of\;Drug}\;\;\times 100\%$$

### Drug Loading Efficiency

The 3.6 mg MSN was uniformly dispersed in 5 mL of ultrapure water as a stock solution. Subsequently, 2.15 mg A_Man was completely dissolved in 2 mL of methanol and slowly dripped into the MSN‐containing aqueous solution under continuous stirring. After 24 h of stirring with gradual evaporation of methanol, the MSN@A_Man was obtained through centrifugation and drying, yielding 5.44 mg of final product. The drug loading efficiency (LE) was calculated using the following formula: (5.44 mg – 3.6 mg) / 3.6 mg × 100% = 51.11%.

(3) $$LE\;\left(\%,\;w/w\right)=\frac{Mass\;of\;drug\;in\;microspheres}{Mass\;of\;microspheres}\times 100\%$$

The determination of drug loading efficiency in G‐CS@Ang(1‐7) was performed following previously reported methods. For detailed procedures, please refer to the protocol described in the aforementioned publication, which will not be reiterated here.^[^ ^48^ ^]^

### Cell Isolation and Culture Seeding

In this study, NPCs were extracted from four‐week‐old male Sprague‐Dawley rats. After sacrificing the rats using excess carbon dioxide, the rats were disinfected with 75% alcohol for 15 min. Complete nucleus pulposus tissue was extracted by surgical clipping and placed in a clean Petri dish. The collected nucleus pulposus tissue was digested with trypsin (0.25%) for 30 min, then digested with type II collagenase (0.2%) for 4 h. After centrifugation, the precipitate was washed twice with PBS. NPCs were cultured in Dulbecco's Modified Eagle Medium (DMEM)/F‐12 medium containing 10% fetal bovine serum (FBS), 100 µg/ml penicillin, and 100 µg mL^−1^ streptomycin. All cell cultures were maintained in a 5% CO_2_ incubator at 37 °C, The first three cell passages (P1‐P3) were utilized for in vitro studies to ensure optimal cell viability and phenotypic stability. All experimental procedures were approved by the Ethics Committee of Shandong University Hospital.

Macrophages were also extracted from four‐week‐old male Sprague‐Dawley rats. After sacrifice, the rats were disinfected with 75% alcohol for 15 min, and the complete femur and tibia were surgically clipped out. Both ends of the bones were cut off and placed in a clean petri dish, then the bone marrow cavity was repeatedly rinsed with DMEM. The collected bone marrow was added to DMEM medium containing 10% FBS, 100 µg mL^−1^ penicillin, and 100 µg mL^−1^ streptomycin then transferred to a cell incubator for culture at 37 °C with 5% CO_2_. After 24 h, the initial liquid was changed to absorb the non‐adherent cells, and fresh DMEM medium containing 30 ng mL^−1^ M‐CSF, 10% FBS, 100 U mL^−1^ penicillin, and 100 µg mL^−1^ streptomycin was added. The liquid was changed every two days during cell culture.

After the cells were cultured to 70–80%, IL‐1β (10 ng mL^−1^) or LPS (100 ng mL^−1^) was added according to the experimental design. After incubation for one day, the relevant drugs or materials were added, and incubation was continued for 4–5 days. Finally, the relevant indicators were detected.

### MTT Assay

The initial cell density was set to 4 × 10^4^. After culturing the cells in the extraction medium for the designated number of days (0, 2, 4, 6, and 8 days), the samples were collected and washed three times with PBS to remove non‐adherent cells. Then, 100 µL of 5 mg mL^−1^ MTT solution and 900 µL of DMEM medium were added to each well, followed by incubation at 37 °C for 4 h. After removing all liquid, 1 mL of formazan dissolving solution was added, and the mixture was gently shaken for 10 min. From each sample well, 200 µL of liquid was transferred to a 96‐well plate, and the optical density value at 490 nm was measured using a microplate reader. Each group was sampled and measured three times to ensure accuracy.

### Live/Dead Cell Staining

The experiment was performed using a Calcein/PI Cell Viability/Cytotoxicity Assay Kit (Beyotime, China). Briefly, NPCs and macrophages were cultured in a 24‐well plate with different stimuli for four days. The cells were then stained with calcein‐AM/PI for 30 min. Samples were observed under a fluorescence microscope (Nikon, Ti2‐U, Japan).

### Cell Apoptosis Assessment

An Annexin V‐FITC Apoptosis Detection Kit (Beyotime, China) was used in this experiment. Briefly, NPCs and macrophages were cultured in a six‐well plate with different stimuli for four days. Cells were stained with Annexin V‐FITC to detect apoptotic cells then analyzed using a flow cytometer (FACSCalibur +Sort, BD, USA).

### Macrophage Polarization Flow Cytometry

Macrophages were cultured in a six‐well plate, and the appropriate treatments were applied for four days. Macrophages were scraped off and labelled with APC‐CD11b/c, PE‐CD206, or FITC‐CD86. The labeled cells were detected using a flow cytometer (BD FACS Celesta, BD, USA).

### Real‐Time Quantitative Polymerase Chain Reaction

qPCR was performed to identify the fold changes in gene expression related to macrophage polarization (CD206, ARG‐1, IL‐1β, INOS) and NPC ECM synthesis, degradation (COL‐2α1, ACAN, MMP‐13, ADAMTS‐5), and inflammation (COX‐2, INOS). β‐actin was used as a housekeeping gene. Briefly, NPCs and macrophages were cultured in six‐well plates and subjected to different treatments for four days. Total RNA was extracted from NPCs using TRIzol reagent (Invitrogen, USA) under different experimental conditions. The total RNA concentration was measured using a NanoDrop spectrophotometer (Thermo Fisher Scientific, MA, USA). According to the instructions, RNA from each sample was reverse transcribed into cDNA using Hifair AdvanceFast 1st Strand cDNA Synthesis SuperMix for qPCR (DNA digester plus) (Yeasen, China). qPCR was performed using Hieff qPCR SYBR Green Master Mix (No Rox) (Yeasen, China). According to the amplification curve, the number of cycles (Ct value) required to reach the threshold fluorescence intensity was obtained, and the relative content of the target gene was 2^−ΔΔCt^. The primers used for each gene are listed in **Table** **1**.

**Table 1:** Primers used in qPCR.

Gene	Primer sequence (F, forward; R, reverse; 5′‐3′)
COL‐2α1	F: AGGAGACAGAGGAGAAGCT R: CTTGAGGACCCTGGATTCC
ACAN	F: CACTTTACTCTTGGTCTTTGTG R: AGTGAGTTGTCATGGTCTG
MMP‐13	F: ACCCAGCCCTATCCCTTGAT R: TCTCGGGATGGATGCTCGTA
iNOS	F: CACCTTGGAGTTCACCCAGT R: ACCACTCGTACTTGGGATGC
COX‐2	F: AATCGCTGTACAAGCAGTGG R: GCAGCCATTTCTTTCTCTCC
ADAMTS‐5	F: ACAACCAGCTAGGTGATGAC R: AATGATGCCCACATAAATCCTC
CD206	F: GAGGACTGCGTGGTGATGAA R: CATGCCGTTTCCAGCCTTTC
Arg‐1	F: AAGACAGGGCTACTTTCAGGAC R: ACCTTCCCGTTTCGTTCCAA
IL‐1β	F: CCAGGATGAGGACCCAAGCA R: TCC CGACCATTGCTGTTTCC
β‐actin	F: CTCTGTGTGGATTGGTGGCT R: CGCAGCTCAGTAACAGTCCG

### Western Blot

Western blotting was used to detect the expression of related proteins. NPCs were cultured in six‐well plates at a density of 5 × 10^5^ cells well^−1^. After treatment and culturing for five days, the cells were lysed in RIPA lysis buffer supplemented with a 1% protease inhibitor mixture or a 1% phosphatase inhibitor mixture and homogenized in a tube on ice for 30 min. After centrifugation at 13 500 rpm for 30 min, the supernatant was collected and stored at –80 °C. Before SDS‐PAGE, the protein concentration in the supernatant was measured using a BCA Protein Assay Kit (Beyotime, China). Protein blotting was performed using equal amounts of different protein samples from each individual sample based on the properties of the different antibodies. After transferring the proteins to polyvinylidene fluoride membranes, the membranes were blocked with 5% nonfat milk in Tris‐buffered saline containing 0.05% Tween 20 for 2 h (the phosphorylation antibody was blocked with 5% BSA for 2 h). The membranes were then incubated overnight with anti‐β‐actin (1:50 000, AC026, abclone, China), anti‐ATF4(1:1000, 60035‐1‐Ig, proteintech, China), anti‐Col2α1(1:500, A19308, abclone, China), anti‐Acan (1:1000, A8536, abclone, China), anti‐eIF2α (1:1000, A0764, abclone, China), anti‐MMP13 (1:1000, A1606, abclone, China), anti‐ADAMTS5 (1:1000, A2836, abclone, China), anti‐mTOR (1:5000, 66888‐1‐Ig, proteintech, China), anti‐PI3K (1:1000, CY5224, abways, China), anti‐INOS (1:1000, 18985‐1‐AP, proteintech, China), anti‐COX‐2 (1:1000, 12375‐1‐AP, proteintech, China), anti‐AKT (1:1000, CY5551, abways, China), anti‐PINK1 (1:1000, 23274‐1‐AP, proteintech, China), anti‐Parkin (1:1000, 14060‐1‐AP, proteintech, China), anti‐Phospho‐mTOR (1:2000, 67778‐1‐Ig, proteintech, China), anti‐Phospho‐PI3K (1:1000, CY6427, abways, China), anti‐Phospho‐AKT (1:2000, 66444‐1‐Ig, proteintech, China), and anti‐Phospho‐eIF2α (1:1000, AP0692, abclone, China). anti‐LC3B (1:1000, 14600‐1‐AP, proteintech, China), anti‐SQSTM1 (1:1000, 29503‐1‐AP, proteintech, China), anti‐TOM20 (1:1000, 11802‐1‐AP, proteintech, China), anti‐MFN2 (1:1000, 12186‐1‐AP, proteintech, China), The membrane was incubated with HRP‐conjugated Goat anti‐rabbit IgG (H+L) (1:50 000, AS014, rabbit monoclonal, Abclone, China) and immunoreactive proteins were detected using enhanced chemiluminescence. Images were captured using the Bio‐Rad ChemiDoc Touch Imaging System, and protein band density was analyzed using ImageJ software. The density values obtained from different target proteins on the same blot were normalized to β‐actin loading controls to calculate the final ratios.

### Immunofluorescence (IF)

The cells were inoculated and added to the different treatment groups. NPCs/macrophages were incubated overnight with different primary antibodies (anti‐Col2α1 (1:100, A19308, abclone, China), anti‐MMP13 (1:100, A1606, abclone, China), anti‐INOS (1:100, 18985‐1‐AP, proteintech, China), anti‐PINK1 (1:100, 23274‐1‐AP, proteintech, China), and anti‐Parkin (1:100, 14060‐1‐AP, proteintech, China)) at 4 °C, then fluorescently labeled, and their cytoskeleton and nucleus were stained with appropriate Alexa Fluor 488 labeled secondary antibodies (K1034G‐AF488, Solarbio, China), TRITC Phalloidin (CA1610, Solarbio, China), and DAPI (S2110, Solarbio, China). Slides were then sealed with an anti‐quenching agent. Stained cells were observed and photographed using a confocal laser microscope (Olympus, Olympus SpinSR10, Japan) or luminescence microscope (Nikon, ZCKP02024640, Japan).

### Alcian Blue Staining

Fresh slides were prepared and fixed in MeOH for 5 min, then rinsed with distilled water and air‐dried slightly. Alcian staining solution (Beyotime, China) was added, and the cells were stained for 30 min, then rinsed twice with distilled water for 1 min each. An Alcian counterstaining solution was applied for 30–60 s. Finally, the slides were rinsed with water and observed under a microscope while wet.

### β‐gal Staining

1 mL of β‐galactosidase staining fixative (Beyotime, China) was added to each well and fixed the cells at room temperature for 15 min. After fixation, the fixative was removed and the cells were washed three times with PBS for 3 min each. After removing the PBS, 1 mL of staining working solution was added to each well and incubated the solution overnight at 37 °C. Images were captured using a standard optical microscope.

### Safranin O Staining

Fresh slides were prepared and fixed in MeOH for 5 min, then rinsed with distilled water and air‐dried slightly. Safranin O staining solution (Beyotime, China) was added, and the cells were stained for 30 min then rinsed twice with distilled water for 1 min each. While still wet, images were observed and captured using a microscope.

### JC‐1 Staining and Flow Cytometry

According to the JC‐1 fluorescent probe kit (Beyotime, China), when the mitochondrial membrane potential is high, JC‐1 accumulates in the mitochondrial matrix and forms polymers that emit red fluorescence under a fluorescence microscope. When the mitochondrial membrane potential is low, JC‐1 does not accumulate in the mitochondrial matrix and remains as a monomer, emitting green fluorescence. In this study, changes in the mitochondrial membrane potential were detected based on shifts in fluorescence color.

### Mitochondrial Lysosome Co‐Localization Staining

Mitochondria were labeled using MitoTracker (Beyotime), and lysosomes were labeled using Lysotracker (Beyotime). After imaging, the Pearson correlation coefficient R was calculated to represent the co‐localization correlation between mitochondria and lysosomes.

### ROS Staining

According to the instructions of the ROS assay kit (Beyotime, China), after culturing and treating the NPCs, ROS staining solution was added for staining. Images were captured by confocal microscopy.

### Co‐Culture of Macrophages and NPCs

Macrophages were absorbed and inoculated on the surface of the sample, which was placed in the upper chamber of the model. The upper chamber of the model was transferred into the pore plate containing NPCs, and detection was performed after adding different treatments for culture.

### Construction of the In Vivo Animal Model of IDD

Animal experiments were approved by the Animal Ethics Committee of Qilu Hospital, Shandong University (ethical approval: DWLL‐2023‐140), and all procedures were conducted in accordance with the guidelines of the Animal Ethics Committee. The experiments were performed in a sterile environment using four‐week‐old Sprague‐Dawley rats. The rats were anesthetized with inhaled isoflurane, then a 26G needle was inserted into the center of the intervertebral disc (C3‐4) under X‐ray guidance. The needle was rotated 360° within the disk and held in place for 30 s before removal. The Sham group only penetrated the skin. The IDD group did not inject drugs. After three days, 5 µL of G‐CS@Ang (1‐7), MSN@(A_Man), or G‐CS/MSN was injected into the puncture site of the disc using the syringe. All procedures strictly adhered to sterile requirements and were repeated five times.

### Imagine Evaluation of Animal Experiments

At four and eight weeks postoperatively, MRI and micro‐CT were performed on rat tails. Micro‐CT imaging was performed using a Bruker SkyScan 2211 system (Belgium). Additionally, a 3.0 T MRI system (Intera Achieva, Philips, Netherlands) was used to scan the rat tails. The intervertebral DHI was calculated using ImageJ 1.52k software, according to the formula DHI = 2 × (A + B + C) / (D + E + F + G + H + I), which involves identifying grayscale values corresponding to the intervertebral discs in a blinded manner. The modified Pfirrmann grading system was employed to assess disc degeneration based on disc structure and signal intensity.

### Hydrogel Degradation Experiment

The cy5‐labeled hydrogel was appropriately injected into the intervertebral discs of rats, and the fluorescence intensity changes at the intervertebral discs were detected on days 0, 3, 7, 10, and 14 using a non‐invasive small‐animal live optical imaging system (IVIS Spectrum) to explore hydrogel degradation in the rat model.

### Histological Evaluation of Animal Experiments

The discs were collected four and eight weeks after treatment. The samples were fixed in 4% paraformaldehyde and decalcified slowly and steadily using EDTA (0.5 M, Servicebio, China). After decalcification, the tissues were dehydrated and embedded in paraffin, and the paraffin blocks were cut into 4‐µm slices on the coronal plane. The slices were stained with H&E and Safranin O‐fast green solutions, Sirius red stain, and polarized light scanning (VS200.Olympus, Japan). The degree of disc degeneration was assessed using a histological grading scale based on five categories of disc changes: moderate disc degeneration with a score of 6–11 and severe disc degeneration with a score of 12–15.

### RNA Sequencing

The number of differentially expressed genes in control, IL‐1β, and IL‐1β+A_Man groups, the fold change of NPC inflammation‐related genes, KEGG enrichment of up/downregulated pathways, and Gene Ontology enrichment of cell function were examined by RNA sequencing.

### Statistical Analysis

In this study, statistical analyses were conducted using SPSS 19 (SPSS Science Inc., Chicago, Illinois) and Prism 6.0 (GraphPad Software, La Jolla, CA, USA). All quantitative data were obtained from at least three independent parallel samples and expressed as the mean ± standard deviation. Comparisons between control and treatment groups were performed using a two‐tailed Student's T‐test. For experiments with more than two groups, one‐way ANOVA was applied, followed by Tukey's post‐hoc test. In cases of non‐parametric data or lack of homogeneity of variance, the Kruskal‐Wallis H test was employed, with subsequent pairwise comparisons using the Nemenyi test. A P‐value of less than 0.05 was considered statistically significant.

### Ethical Statement

All animal experiments in this study were performed in accordance with institutional guidelines and approved by the Laboratory Animal Centre of Qilu Hospital of Shandong University (DWLL‐2023–139). The study of human tissue was approved by the institutional review board of Qilu Hospital of Shandong University (KYLL‐2022(ZM)−1051).

## Conflict of Interest

The authors declare no conflict of interest.

## Author Contributions

X.K., R.H., and P.Z. contributed equally to this work. X.K., R.H., P.Z., L.C., L.J., C.L., and Z.W. designed the research. X.K., R.H., P.Z., L.L., Q.L., K.S., and H.G. performed the experiments. X.K., J.Z., and S.L. analyzed the data. X.K. and R.H. wrote this paper.

## Supporting information



Supporting Information

## Data Availability

The data that support the findings of this study are available from the corresponding author upon reasonable request.

## References

[1] G. B. D. Disease , I. Injury , C. Prevalence , Lancet 2017, 392, 1789.

[2] T. Oichi , Y. Taniguchi , Y. Oshima , S. Tanaka , T. Saito , J. O. R. Spine 2020, 3, 1076. 10.1002/jsp2.1076 PMC7084053 32211588

[3] a) X. Du , K. Liang , S. Ding , H. Shi , Biomedicines 2023, 11, 11092467; 10.3390/biomedicines11092467 PMC10525468 37760908

[4] a) F. J. Lyu , H. Cui , H. Pan , K. M. Cheung , X. Cao , J. C. Iatridis , Z. Zheng , Bone Res. 2021, 9, 7; 33514693 10.1038/s41413-020-00125-x PMC7846842

[5] L. Frapin , J. Clouet , V. Delplace , M. Fusellier , J. Guicheux , C. L. Visage , Adv. Drug Deliv. Rev. 2019, 149, 49. 31445063 10.1016/j.addr.2019.08.007

[6] Y. Xu , J. Lv , F. Liu , J. Wang , Y. Liu , C. Kong , Y. Li , N. Shen , Z. Gu , Z. Tang , X. Chen , Adv. Mater. 2024, 36, 2312493. 10.1002/adma.202312493 38444177

[7] a) P. Feng , Y. Che , C. Gao , L. Zhu , J. Gao , N. V. Vo , Front. Immunol. 2023, 14, 1155746; 37122738 10.3389/fimmu.2023.1155746 PMC10140429

[8] a) R. A. S. Santos , W. O. Sampaio , A. C. Alzamora , D. Motta‐Santos , N. Alenina , M. Bader , M. J. Campagnole‐Santos , Physiol. Rev. 2018, 98, 505; 29351514 10.1152/physrev.00023.2016 PMC7203574

[9] G. Gu , B. Zhu , J. Ren , X. Song , B. Fan , H. Ding , J. Shang , H. Wu , J. Li , H. Wang , J. Li , Z. Wei , S. Feng , Cell Biosci. 2023, 13, 23. 36739421 10.1186/s13578-023-00967-y PMC9899400

[10] a) L. G. Chen , L. L. Yang , C. C. Wang , Food Chem. Toxicol. 2008, 46, 688; 18029076 10.1016/j.fct.2007.09.096

[11] J. Chen , M. Bian , L. Pan , H. Yang , J. Appl. Toxicol. 2022, 42, 1467. 35187677 10.1002/jat.4306

[12] N. Yuan , K. Shao , S. Huang , C. Chen , Int. J. Biol. Macromol. 2023, 240, 124321. 37019198 10.1016/j.ijbiomac.2023.124321

[13] Y. Chen , W. Sheng , J. Lin , C. Fang , J. Deng , P. Zhang , M. Zhou , P. Liu , J. Weng , F. Yu , D. Wang , B. Kang , H. Zeng , ACS Appl. Mater. Interfaces 2022, 14, 7592. 35119809 10.1021/acsami.1c21260

[14] J. Y. Oh , G. Yang , E. Choi , J. H. Ryu , Biomater. Sci. 2022, 10, 1448. 35229845 10.1039/d2bm00010e

[15] Y. Wang , Q. Zhao , N. Han , L. Bai , J. Li , J. Liu , E. Che , L. Hu , Q. Zhang , T. Jiang , S. Wang , Nanomedicine 2015, 11, 313. 25461284 10.1016/j.nano.2014.09.014

[16] S. M. Abuzar , S. M. Hyun , J. H. Kim , H. J. Park , M. S. Kim , J. S. Park , S. J. Hwang , Int. J. Pharm. 2018, 538, 1. 29278733 10.1016/j.ijpharm.2017.12.041

[17] S. Tian , X. Chen , W. Wu , H. Lin , X. Qing , S. Liu , B. Wang , Y. Xiao , Z. Shao , Y. Peng , Exp. Mol. Med. 2024, 56, 408. 38316963 10.1038/s12276-024-01168-4 PMC10907345

[18] K. Pakos‐Zebrucka , I. Koryga , K. Mnich , M. Ljujic , A. Samali , A. M. Gorman , EMBO Rep. 2016, 17, 1374. 27629041 10.15252/embr.201642195 PMC5048378

[19] A. F. Zyryanova , K. Kashiwagi , C. Rato , H. P. Harding , A. Crespillo‐Casado , L. A. Perera , A. Sakamoto , M. Nishimoto , M. Yonemochi , M. Shirouzu , T. Ito , D. Ron , Mol Cell 2021, 81, 88. 33220178 10.1016/j.molcel.2020.10.031 PMC7837216

[20] G. Z. Zhang , Y. J. Deng , Q. Q. Xie , E. H. Ren , Z. J. Ma , X. G. He , Y. C. Gao , X. W. Kang , Clin. Chim. Acta 2020, 508, 33. 32348785 10.1016/j.cca.2020.04.016

[21] L. Xu , H. Gao , Y. Deng , X. Liu , W. Zhan , X. Sun , J. J. Xu , G. Liang , Biosens. Bioelectron. 2024, 255, 116207. 38554575 10.1016/j.bios.2024.116207

[22] X. Wu , Y. Song , W. Liu , K. Wang , Y. Gao , S. Li , Z. Duan , Z. Shao , S. Yang , C. Yang , Cell Death Discov. 2017, 3, 16107. 28149534 10.1038/cddiscovery.2016.107 PMC5280875

[23] R. Lesmana , S. Tandean , A. Christoper , A. A. Suwantika , N. Wathoni , R. Abdulah , J. Fearnley , V. Bankova , F. Zulhendri , Biomed. Pharmacother. 2024, 175, 116745. 38761422 10.1016/j.biopha.2024.116745

[24] L. Kma , T. J. Baruah , Biotechnol. Appl. Biochem. 2022, 69, 248. 33442914 10.1002/bab.2104

[25] C. Guo , Y. Liu , Z. Zhao , Y. Wu , Q. Kong , Y. Wang , J. Control Release 2024, 365, 1004. 38128882 10.1016/j.jconrel.2023.12.029

[26] a) F. Tao , Y. Cheng , X. Shi , H. Zheng , Y. Du , W. Xiang , H. Deng , Carbohydr Polym 2020, 230, 115658; 31887899 10.1016/j.carbpol.2019.115658

[27] Y. H. Cheng , S. H. Yang , F. H. Lin , Biomaterials 2011, 32, 6953. 21774981 10.1016/j.biomaterials.2011.03.065

[28] M. Sarem , F. Moztarzadeh , M. Mozafari , Carbohydr. Polym. 2013, 93, 635. 23499106 10.1016/j.carbpol.2012.11.099

[29] a) S. Liu , K. Li , Y. He , S. Chen , W. Yang , X. Chen , S. Feng , L. Xiong , Y. Peng , Z. Shao , Adv. Sci. 2024, 11, 2400749; 10.1002/advs.202400749 PMC11165536 38554394

[30] a) G. J. Grosicki , B. B. Barrett , D. A. Englund , C. Liu , T. G. Travison , T. Cederholm , A. Koochek , A. von Berens , T. Gustafsson , T. Benard , K. F. Reid , R. A. Fielding , J. Frailty Aging 2020, 9, 57; 32150215 10.14283/jfa.2019.30 PMC12275783

[31] a) C. Franceschi , M. Bonafe , S. Valensin , F. Olivieri , M. De Luca , E. Ottaviani , G. De Benedictis , Ann. N. Y. Acad. Sci. 2000, 908, 244; 10911963 10.1111/j.1749-6632.2000.tb06651.x

[32] a) T. Fulop , J. M. Witkowski , F. Olivieri , A. Larbi , Semin. Immunol. 2018, 40, 17; 30287177 10.1016/j.smim.2018.09.003

[33] M. Locati , G. Curtale , A. Mantovani , Annu. Rev. Pathol. 2020, 15, 123. 31530089 10.1146/annurev-pathmechdis-012418-012718 PMC7176483

[34] B. Chazaud , Trends Immunol. 2020, 41, 481. 32362490 10.1016/j.it.2020.04.006

[35] Y. Takayama , T. Ando , J. Ichikawa , H. Haro , Sci. Rep. 2018, 8, 11320. 30054581 10.1038/s41598-018-29669-z PMC6063965

[36] a) M. Szaruga , D. A. Janssen , C. de Miguel , G. Hodgson , A. Fatalska , A. P. Pitera , A. Andreeva , A. Bertolotti , Nat. Commun. 2023, 14, 5535; 37684277 10.1038/s41467-023-40823-8 PMC10491595

[37] J. C. Valdoz , B. C. Johnson , D. J. Jacobs , N. A. Franks , E. L. Dodson , C. Sanders , C. G. Cribbs , P. M. Van Ry , Int. J. Mol. Sci. 2021, 22, 12690. 34884495 10.3390/ijms222312690 PMC8657545

[38] N. Levi , N. Papismadov , I. Solomonov , I. Sagi , V. Krizhanovsky , FEBS J. 2020, 287, 2636. 32145148 10.1111/febs.15282

[39] E. S. White , Ann. Am. Thorac. Soc. 2015, 12, S30. 25830832 10.1513/AnnalsATS.201406-240MG PMC4430981

[40] T. Pan , R. Chen , D. Wu , N. Cai , X. Shi , B. Li , J. Pan , Int. Immunopharmacol. 2017, 52, 156. 28915439 10.1016/j.intimp.2017.08.021

[41] K. Sun , J. Luo , J. Guo , X. Yao , X. Jing , F. Guo , Osteoarthrit. Cartil. 2020, 28, 400. 10.1016/j.joca.2020.02.027 32081707

[42] Y. S. Li , F. J. Zhang , C. Zeng , W. Luo , W. F. Xiao , S. G. Gao , G. H. Lei , Joint Bone Spine 2016, 83, 143. 26453105 10.1016/j.jbspin.2015.06.009

[43] Z. Xu , X. Han , D. Ou , T. Liu , Z. Li , G. Jiang , J. Liu , J. Zhang , Appl. Microbiol. Biotechnol. 2020, 104, 575. 31832711 10.1007/s00253-019-10257-8

[44] X. Xu , D. Wang , C. Zheng , B. Gao , J. Fan , P. Cheng , B. Liu , L. Yang , Z. Luo , Theranostics 2019, 9, 2252. 31149042 10.7150/thno.30658 PMC6531300

[45] Y. Song , H. Liang , G. Li , L. Ma , D. Zhu , W. Zhang , B. Tong , S. Li , Y. Gao , X. Wu , Y. Zhang , X. Feng , K. Wang , C. Yang , Autophagy 2024, 20, 809. 37876250 10.1080/15548627.2023.2274205 PMC11062375

[46] Y. Lu , Z. Li , S. Zhang , T. Zhang , Y. Liu , L. Zhang , Theranostics 2023, 13, 736. 36632220 10.7150/thno.79876 PMC9830443

[47] M. Onishi , K. Yamano , M. Sato , N. Matsuda , K. Okamoto , EMBO J. 2021, 40, 104705. 10.15252/embj.2020104705 PMC7849173 33438778

[48] N. Farhadian , M. Godiny , S. Moradi , H. Azandaryani , M. Shahlaei , Mater. Sci. Eng. C Mater. Biol. Appl. 2018, 92, 540. 30184780 10.1016/j.msec.2018.07.002

