# Supplementary material for: Modulating Macrophage–Nucleus Pulposus Cell Crosstalk via Sequential Drug Delivery Attenuates Disc Degeneration
Source: Adv Sci (Weinh). 2025 Jun 30;12(36):e07178. doi: 10.1002/advs.202507178 (PMC12463071; doi:10.1002/advs.202507178)
Supplement: Supplementary file 1 — Supporting Information [file ADVS-12-e07178-s001.docx]

**Modulating Macrophage–Nucleus Pulposus Cell Crosstalk via Sequential Drug Delivery Attenuates Disc Degeneration**

**Supplementary Data**

**Supplementary Experimental Procedures**

**Procedure of immunohistochemical experiment in paraffin section**

1. Dewaxing paraffin sections to water: Put the sections into xylene Ⅰ 15min-xylene Ⅱ 15min-anhydrous ethanol Ⅰ 5min-anhydrous ethanol Ⅱ5min-85% alcohol 5min-75% alcohol 5min-distilled water in turn.

2. Antigen repair: Tissue sections are placed in a repair box filled with citric acid antigen repair buffer (PH6.0) for antigen repair in a microwave oven. Medium fire for 5 minutes, during this process should prevent excessive evaporation of buffer, do not dry. After natural cooling, the slide was placed in PBS (PH7.4) and washed by shaking on the decolorizing shaker for 3 times, 5min each time.

3. Blocking: The section was put into 3% hydrogen peroxide solution and incubated at room temperature for 25min away from light to block endogenous peroxidase. After that, the slide was placed in PBS (PH7.4) and washed by shaking on the decolorizing table for 3 times, 5min each time.

4. Draw circles: After the section is slightly dried, draw circles around the tissue with a tissue pen.

5. Serum closure: add 3%BSA in the circle and incubate at room temperature for 30min.

6. Add primary antibody: gently shake off the sealing solution, add PBS to the section according to a certain proportion of primary antibody, and place the section flat in a wet box at 4°C for overnight incubation.

7. Add secondary antibody: The slide is placed in PBS (PH7.4) and washed by shaking on the decolorizing table for 3 times, 5min each time. After the slices were slightly dried, the tissue covered by the second antibody of the corresponding species of the first antibody was added to the ring and incubated at room temperature for 50min away from light.

8. DAB color development and restaining nuclei: The slide was placed in PBS (PH7.4) and washed by shaking on the decolorizing table for 3 times, 5min each time. After the sections were slightly dried, DAB color developing agent was added in the circle, and the color development was controlled by microscope. After the color development is complete, rinse with distilled water or tap water, hematoxylin restaining, 1% hydrochloric acid alcohol differentiation (about 1s), tap water rinse, ammonia water back blue, running water rinse.

9. Dewatering and sealing: Put the slices into 75% alcohol 5min-85% alcohol 5min- anhydrous ethanol Ⅰ5min - anhydrous ethanol Ⅱ5min - xylene Ⅰ5min to dehydrate transparent, take the slices out of xylene to dry slightly, and seal them with neutral gum.

**Double-label + triple-label immunofluorescence in paraffin sections (TSA method)**

1. Dewaxing paraffin sections to water: Put the sections into xylene Ⅰ 15min-xylene Ⅱ 15min-anhydrous ethanol Ⅰ 5min-anhydrous ethanol Ⅱ5min-85% alcohol 5min-75% alcohol 5min-distilled water in turn.

2. Antigen repair: The tissue sections were placed in a pressure cooker filled with citric acid repair solution (PH6.0), the pressure cooker was placed on the induction cooker, heated until the gas came out and started timing, timing for 2min, and placed in the tap water to cool. After the pressure in the pressure cooker was reduced to natural pressure, the lid of the high-pressure cooker was opened and cooled naturally. After that, the slide was placed in PBS (PH7.4) and washed by shaking on the decolorizing table for 3 times, 5min each time.

3. Blocking: The section was put into 3% hydrogen peroxide solution and incubated at room temperature for 25min away from light to block endogenous peroxidase. After that, the slide was placed in PBS (PH7.4) and washed by shaking on the decolorizing table for 3 times, 5min each time.

4. Draw circles: After the section is slightly dried, draw circles around the tissue with a tissue pen.

5. Serum closure: Add 10% goat serum in the circle and incubate at room temperature for 30min.

6. Add primary antibody: gently shake off the sealing liquid, add the prepared primary antibody to the slice, and place the slice flat in a wet box at 4°C for overnight incubation. (Add a small amount of water to the wet box to prevent the antibody from evaporating)

7. Add secondary antibody: The slide is placed in PBS (PH7.4) and washed by shaking on the decolorizing table for 3 times, 5min each time. After the slices were slightly dried, the tissue was covered with the secondary antibody of the corresponding species of the primary antibody (HRP enzyme label), and incubated at room temperature for 50min away from light.

8. Add TSA reagent: The slide is placed in PBS (PH7.4) and washed by shaking on the decolorizing table for 3 times, 5min each time. The tissue was added with tyramine salt-CY3 (freshly prepared before use, PBST configuration containing 0.003%H2O2) and incubated at room temperature for 20min. Then wash with PBS three times, 5min each time.

9. Microwave repair: Put the slide in 1× citric acid repair solution, repair it with microwave oven at high heat for 6min, and cool it to room temperature naturally.

10. Draw circles: After the section is slightly dried, draw circles around the tissue with a tissue pen.

11. Serum closure: add 10% goat serum in the circle and incubate for 10min at room temperature.

12. Add primary antibody: gently shake off the sealing liquid, add the prepared primary antibody to the slice, and place the slice flat in a wet box at 4°C for overnight incubation. (Add a small amount of water to the wet box to prevent the antibody from evaporating)

13. Add secondary antibody: The slide is placed in PBS (PH7.4) and washed by shaking on the decolorizing table for 3 times, 5min each time. After the slices were slightly dried, the tissue was covered with the secondary antibody of the corresponding species of the primary antibody (HRP enzyme label), and incubated at room temperature for 50min away from light.

14. Add TSA reagent: The slide is placed in PBS (PH7.4) and washed by shaking on the decolorizing table for 3 times, 5min each time. Add tyramine salt-488 to the tissue (before use, use the PBST configuration containing 0.003%H2O2) and incubate at room temperature for 20min. Then wash with PBS three times, 5min each time.

15. DAPI restaining nuclei: The slide was placed in PBS (PH7.4) and washed by shaking on the decolorizing shaker for 3 times, 5min each time. After the slices were slightly dried, DAPI dye was added into the circle and incubated for 10min at room temperature away from light.

16. Sealing: The slide is placed in PBS (PH7.4) and washed by shaking on the decolorizing table for 3 times, 5min each time. The slices were slightly dried and sealed with anti-fluorescence quenching tablets.

17. Microscopic photography: section under the fluorescence microscope observation and acquisition of images.

.
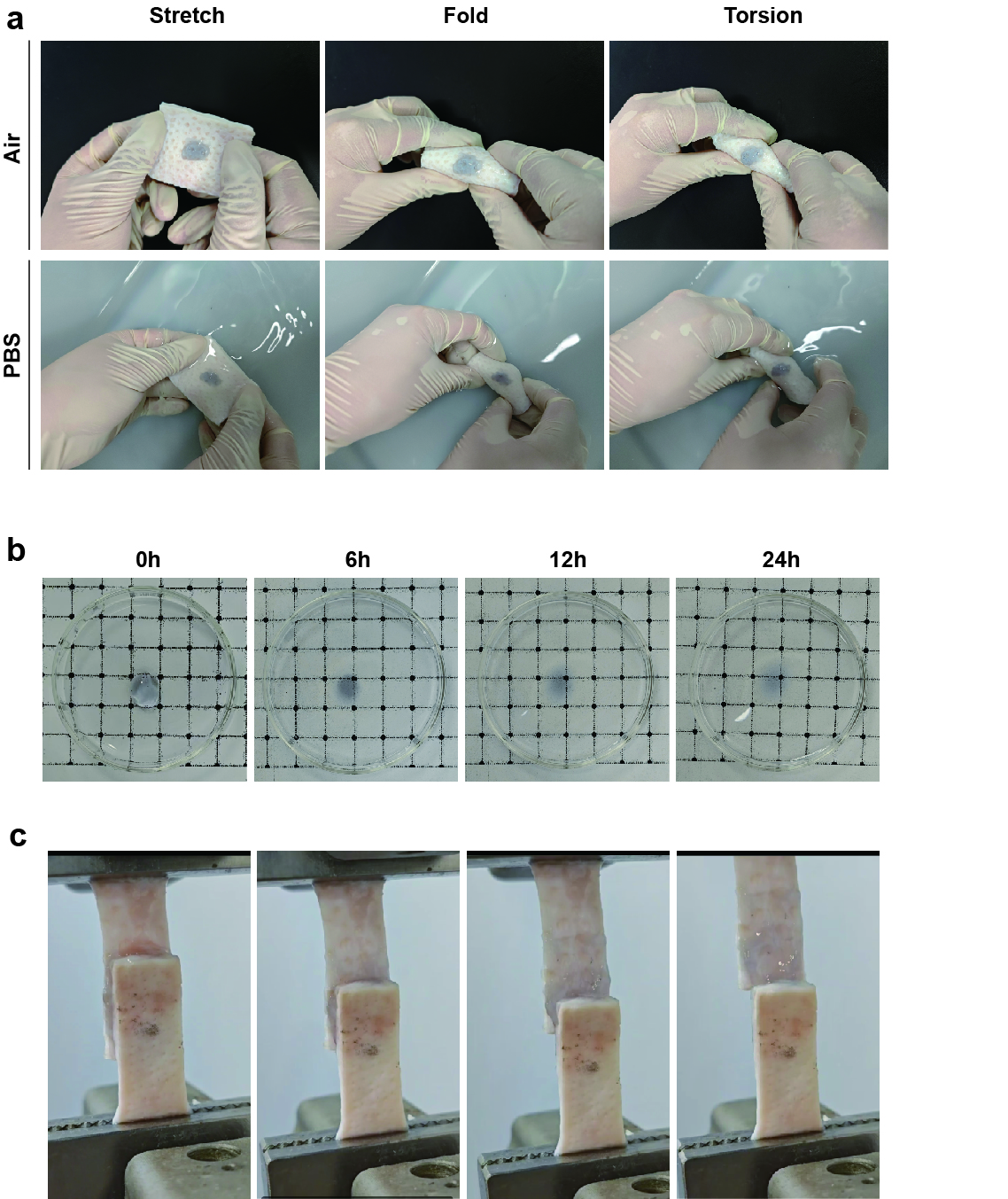


Figure S1. (a) The hydrogel exhibits robust adhesion performance when subjected to mechanical stresses, including twisting and folding, maintaining its adhesive properties in ambient air and PBS environments. (b) The hydrogel demonstrates remarkable swelling resistance in PBS over varying periods (0h, 6h, 12h, 24h). (c) The tensile stress of the hydrogel was evaluated under adhesion stress testing conditions, with porcine skin serving as the substrate.


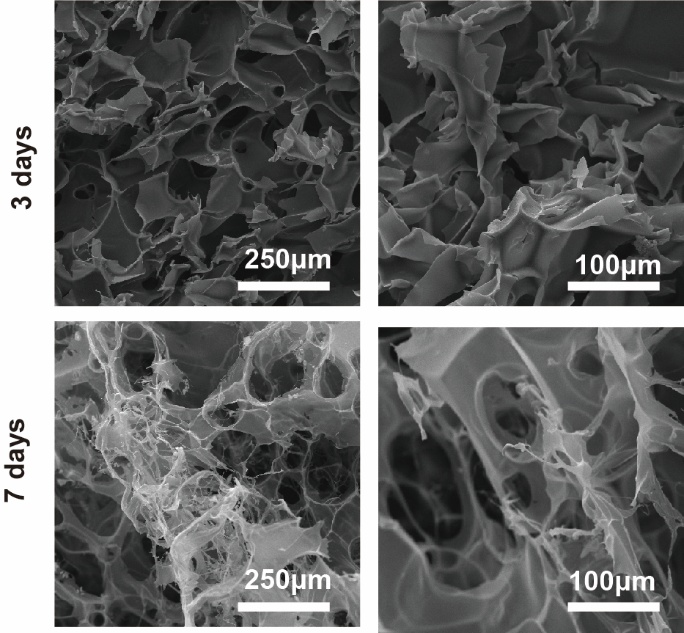


Figure S2. SEM images of G-CS degradation after 3 and 7 days (scale bar: 250μm, 100μm).





Figure S3. The release curve of A_Man in MSN@A_Man


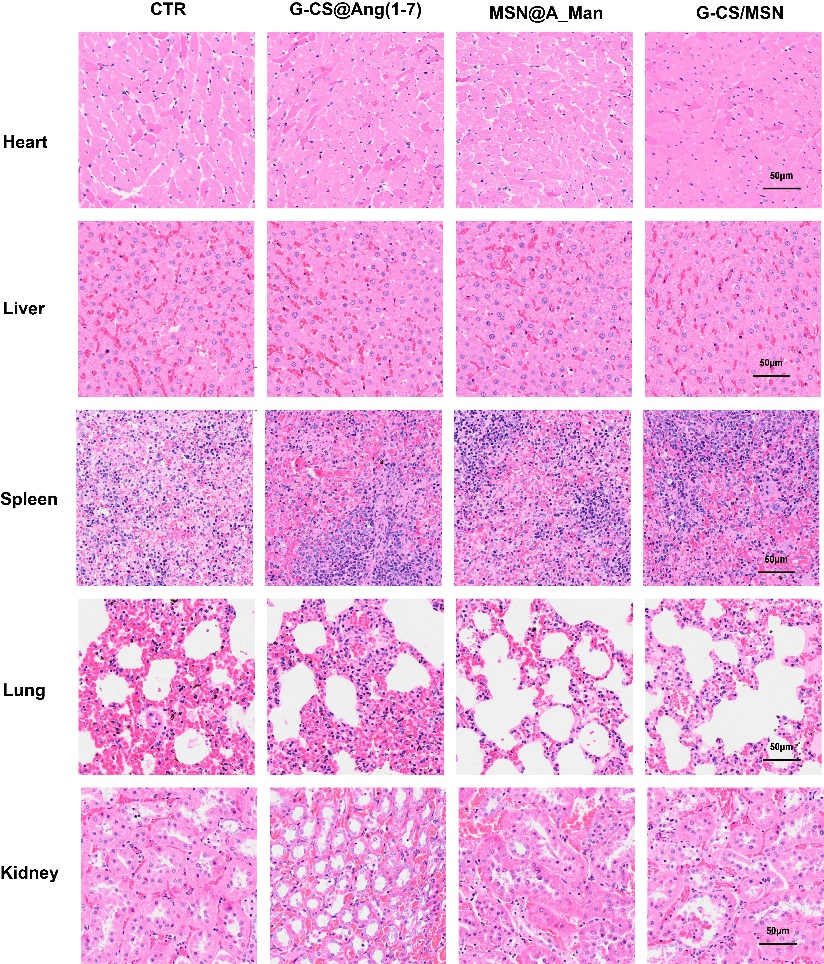


Figure S4. H&E staining of heart, liver, spleen, lung and kidney of different treated animals (n=5, scale bar: 50μm).


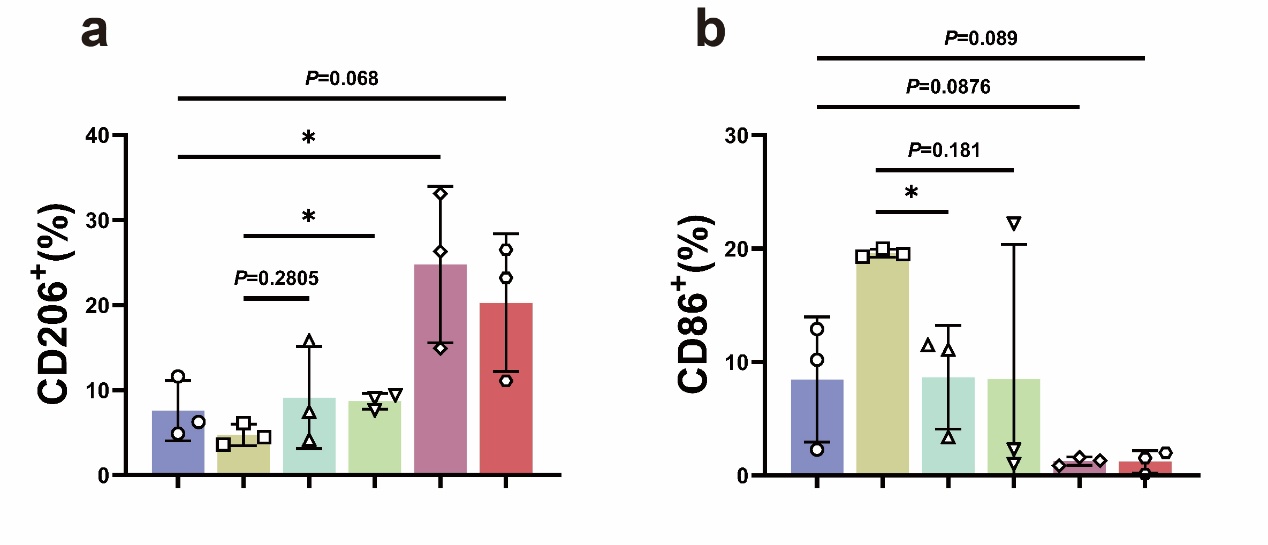
Figure S5. The polarization of macrophages was detected by flow cytometry (n=3, *P< 0.05, **P< 0.01, ***P< 0.001).


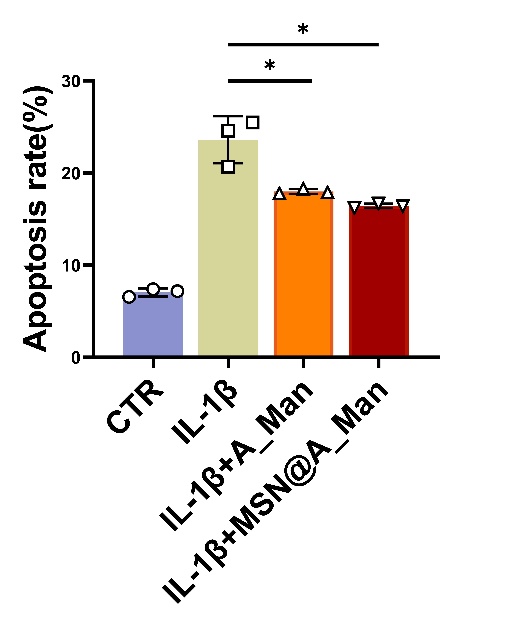


Figure S6. Apoptosis analysis of NPCs (n=3, *P< 0.05, **P< 0.01, ***P< 0.001).


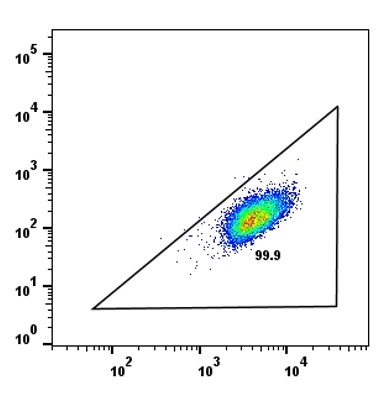


Figure S7. Flow cytometry of JC-1, CCCP^+^.


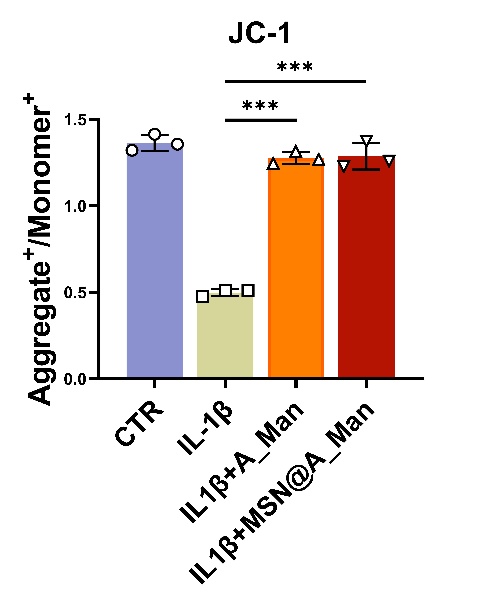


Figure S8. Statistical analysis of JC-1 (n=3, *P< 0.05, **P< 0.01, ***P< 0.001).


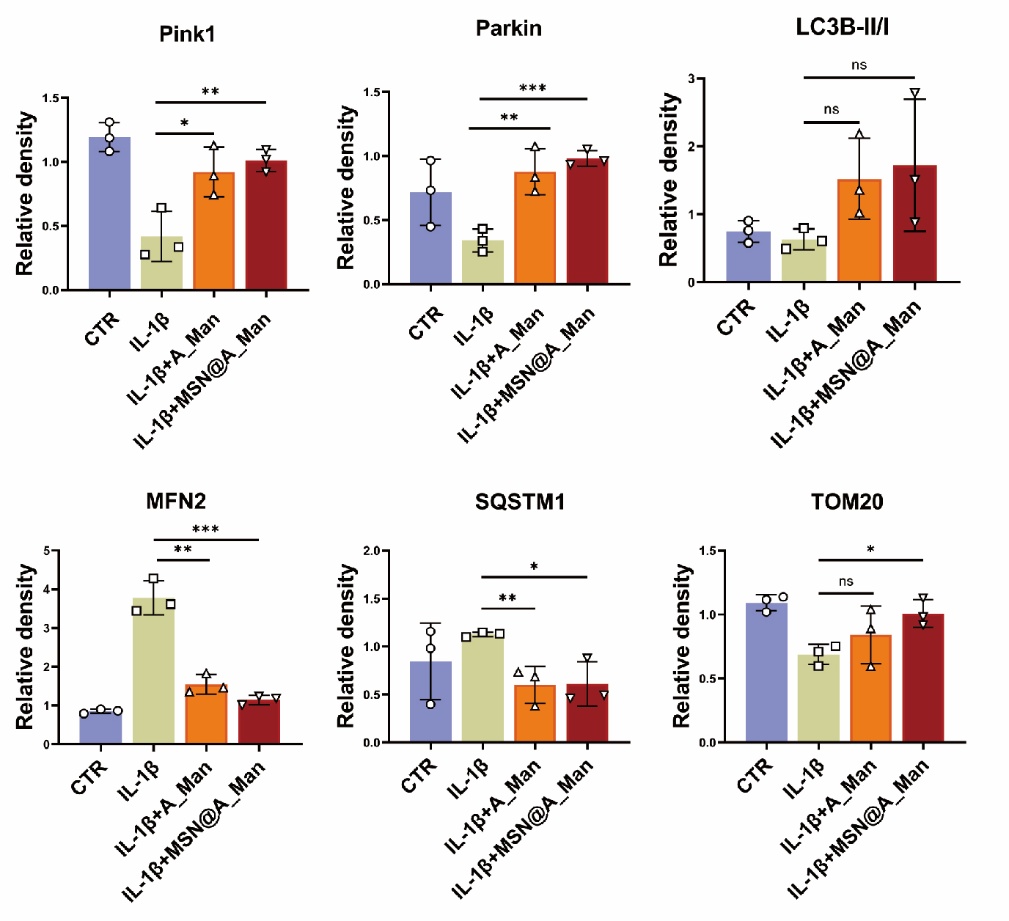


Figure S9. Western-Blot results statistical analysis of Pink1, Parkin, MFN2, SQSTM1, LC3B, TOM20 (n=3, *P< 0.05, **P< 0.01, ***P< 0.001).


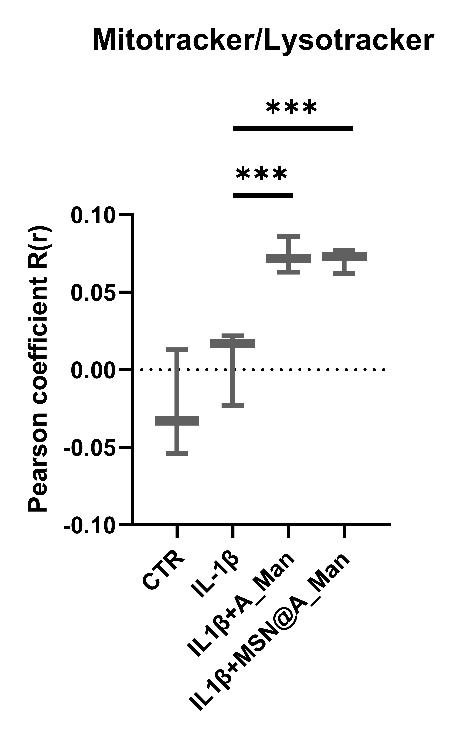


Figure S10. Statistical analysis of colocation area (n=3, *P< 0.05, **P< 0.01, ***P< 0.001).

**
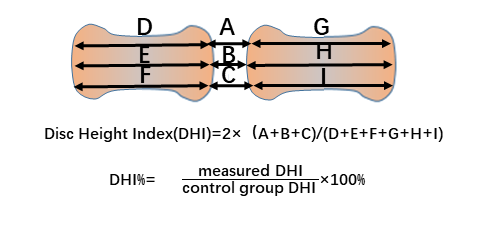
**

Figure S11. DHI calculation method.

Table 1. Pfirrmann disc degeneration score

|  | I | II | III | IV | V |
| --- | --- | --- | --- | --- | --- |
| Structure | Homogeneous, bright white | Inhomogeneous with or without horizontal bands | Inhomogeneous, gray | Inhomogeneous, gray to black | Inhomogeneous, black |
| Distinction of Nucleus and Anulus | Clear | Clear | Unclear | Lost | Lost |
| Signal Intensity | Hyperintense, isointense  to cerebrospinal fluid | Hyperintense, isointense  to cerebrospinal fluid | Intermediate | Intermediate to hypointense | Hypointense |
| Height of Interverteb ral Disc | Normal | Normal | Normal to slightly decreased | Normal to moderately decreased | Collapsed disc space |

| Table 2. Histological grade scale of intervertebral disc | |
| --- | --- |
| I. Nucleus pulposus morphology | II. Nucleus pulposus cellularity |
| Grade: | Grade: |
| 0: round shape, nucleus pulposus > 75% of disc area | 0: stellar-shaped cells |
| 1: round shape, nucleus pulposus = 50% -75% of disc area | 1: most stellar-shaped cells with some round cells |
| 2: nucleus pulposus = 25% -50% of disc area | 2: most round cells with some stellar- shaped cells |
| 3: nucleus pulposus < 25% of disc area | 3: round-shaped cells |
| III. Annulus fibrosus morphology | IV. Annulus fibrosus cellularity |
| Grade: | Grade: |
| 0: well-organized lamellae with no ruptures | 0: fibroblasts > 90% of the cells |
| 1: ruptured fibers < 25% of the annulus fibrosus | 1: fibroblasts = 75% - 90% of the cells |
| 2: ruptured fibers = 25% - 50% of the annulus fibrosus | 2: fibroblasts = 25% - 75% of the cells |
| 3: ruptured fibers > 50% of the annulus fibrosus | 3: fibroblasts < 25% of the cells |
| V. Border between the nucleus pulposus and annulus fibrosus | |
| Grade: |  |
| 0: normal, without any interruption |  |
| 1: minimal interruption |  |
| 2: moderate interruption |  |
| 3: severe interruption |  |
